# ConDoR: tumor phylogeny inference with a copy-number constrained mutation loss model

**DOI:** 10.1186/s13059-023-03106-5

**Published:** 2023-11-30

**Authors:** Palash Sashittal, Haochen Zhang, Christine A. Iacobuzio-Donahue, Benjamin J. Raphael

**Affiliations:** 1 Department of Computer Science, Princeton University, NJ, USA; 2 Gerstner Sloan Kettering Graduate School of Biomedical Sciences, Memorial Sloan Kettering Cancer Center, NY, USA; 3 Human Oncology and Pathogenesis Program, Memorial Sloan Kettering Cancer Center, NY, USA; 4 David M. Rubenstein Center for Pancreatic Cancer Research, Memorial Sloan Kettering Cancer Center, NY, USA; 5 Department of Pathology and Laboratory Medicine, Memorial Sloan Kettering Cancer Center, NY, USA

**Keywords:** Cancer, Intra-tumor heterogeneity, Tumor phylogeny, Single-cell DNA sequencing, Dollo model

## Abstract

A tumor contains a diverse collection of somatic mutations that reflect its past evolutionary history and that range in scale from single nucleotide variants (SNVs) to large-scale copy-number aberrations (CNAs). However, no current single-cell DNA sequencing (scDNA-seq) technology produces accurate measurements of both SNVs and CNAs, complicating the inference of tumor phylogenies. We introduce a new evolutionary model, the *constrained* *k-Dollo model*, that uses SNVs as phylogenetic markers but constrains losses of SNVs according to clusters of cells. We derive an algorithm, ConDoR, that infers phylogenies from targeted scDNA-seq data using this model. We demonstrate the advantages of ConDoR on simulated and real scDNA-seq data.

**Supplementary Information:**

The online version contains supplementary material available at 10.1186/s13059-023-03106-5.

## Background

Cancer is an evolutionary process in which somatic mutations across all genomic scales―ranging from single nucleotide variants (SNVs) to large-scale copy number aberrations (CNAs)―accumulate in a population of cells. This process results in a heterogeneous tumor with subpopulations of cells, called *clones*, with distinct genomes. Reconstruction of the evolutionary history of cancer clones, known as a tumor phylogeny, from genomic sequencing data of the cells in a tumor is crucial for understanding cancer progression and developing effective therapies for treatment [1–4].

Early cancer sequencing projects performed bulk sequencing of tumor samples and thus measured somatic mutations from a mixture of thousands or millions of cells. Tumor phylogeny inference from this data is complicated since it requires deconvolution of the data, i.e., simultaneous inference of the tumor clones and their proportions in the mixture [5–11].

Recent developments in single-cell DNA sequencing (scDNA-seq) allow parallel sequencing of thousands of individual cells from a tumor  [2, 12–15], alleviating the need for such deconvolution. However, tumor phylogeny inference from this data remains challenging since current scDNA-seq technologies are error-prone and produce data with missing information. As such, phylogeny inference using scDNA-seq data involves correcting these errors and imputing the missing data under some evolutionary model [2, 16].

Multiple evolutionary models have been used to construct tumor phylogenies from scDNA-seq data. Early works [17–20] used SNVs as evolutionary markers, and relied on the *infinite-sites model* [21] which states that an SNV can be gained only once and never be subsequently lost in the phylogeny. While the same SNV occurring independently more than once is rare [22], loss of SNVs due to copy-number deletions is common in cancer [23]. To account for these losses, other works [24–27] use some variant of the *k-Dollo model* [28], in which a mutation can be gained at most once but may be lost at most *k* times during the course of the evolution, where *k* is a user-defined integer. Several methods [29, 30] employ an even more permissive model, the *finite-sites model* [31], which allowed mutations to be gained and lost multiple times.

A major limitation of the aforementioned models and methods is that they do not utilize any information about CNAs, which can often also be derived from scDNA-seq data. This limitation is addressed in methods such as SCARLET [32], BiTSC$^2$ [33], and COMPASS [34] which incorporate copy-number information during phylogeny inference. SCARLET introduced a novel loss-supported Dollo model that requires the copy-number profile of each cell and the copy-number phylogeny as input. BiTSC$^2$ and COMPASS, on the other hand, construct a joint phylogeny with both SNV and CNA events. However, these methods rely heavily on accurate and simultaneous identification of SNVs and CNAs on the same set of cells, which is challenging with the current scDNA-seq technologies [35].

Current scDNA-seq technologies fall into one of two classes with different capabilities from measuring CNAs and SNVs. First, *whole genome* scDNA-seq technologies yield data with roughly uniform coverage of the whole genome but with low depth at any particular locus, making it suitable for detection of larger CNAs in single-cells but not SNVs [12, 14, 36]. In contrast, *targeted* scDNA-seq technologies sequence specific regions of the genome, typically comprising of cancer-related genes, with high depth allowing accurate identification of SNVs but not of CNAs [13, 15, 24, 37]. For example, the Mission Bio Tapestri platform [38, 39] performs high-coverage sequencing ($\sim 50\times$ coverage) of hundreds of amplicons from thousands of cells. While precise identification of CNAs in each cell using such targeted scDNA-seq data is challenging, clustering of cells based on their copy-number profiles is a much simpler task. However, no existing evolutionary model utilizes such clustering information.

**Fig. 1:**
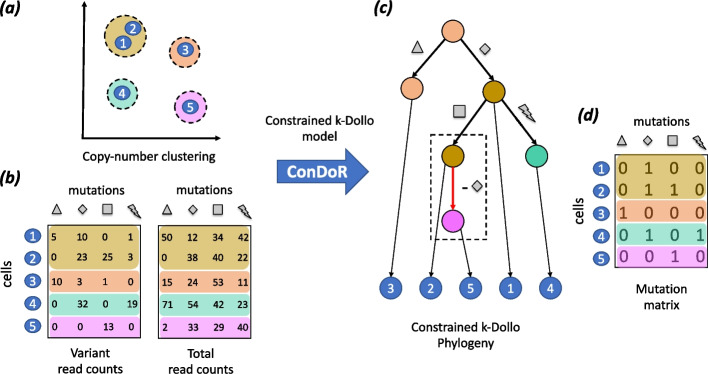
Overview of the ConDoR algorithm. ConDoR takes as input: **a** a clustering of cells based on copy-number profiles and **b** the observed variant and total read counts from scDNA-seq data. ConDoR employs the constrained *k*-Dollo model to construct the **c** constrained *k*-Dollo phylogeny with mutation losses (dashed box) allowed only between cells from distinct copy-number clusters and the **d** inferred mutation matrix

Here, we introduce a new evolutionary model, the *constrained* *k-Dollo model*, and an algorithm ConDoR (Constrained Dollo Reconstruction) that computes phylogenetic trees from read count data using this model (Fig. 1). In the *constrained* *k-Dollo model*, an SNV occurs only once on the phylogeny but may be lost up to *k* times, as long as these losses conform to a given copy-number clustering of cells. The key idea underpinning the constrained *k*-Dollo model is that, since loss of SNVs predominantly occurs due to CNAs, we allow loss of an SNV only between cells that have distinct copy-number profiles. Importantly, the constrained *k*-Dollo model generalizes both the infinite-sites and the *k*-Dollo models. Additionally, we model the loss of single nucleotide polymorphisms (SNPs), i.e., germline variants present in normal cells, that can be informative during phylogeny inference, while most existing methods only focus on somatic variants (SNVs) [17, 20, 25, 32].

We show that ConDoR outperforms existing tumor phylogeny inference methods on simulated and real targeted scDNA-seq data, including Mission Bio Tapestri data from multiple regions of a pancreatic tumor and whole-exome sequencing of a metastatic colorectal cancer [37]. In both cases, ConDoR yields a more plausible phylogeny compared to existing methods, and provides insights into the spatial evolution and metastatic spread of these tumors.

## Results

### Constrained *k*-Dollo model

We propose a new model, the *constrained* *k-Dollo model*, that integrates information about SNVs, SNPs and CNAs on the same set of cells during phylogeny inference. Our model incorporates CNAs via a clustering of cells, where all cells in the same cluster have the same copy-number profile (the copy number of all loci across the genome). In other words, each cluster corresponds to a *copy-number clone*. We will refer to this clustering as the *copy-number clustering*.

Suppose we measure *m* SNVs and SNPs in *n* cells from a tumor. In the following, we collectively refer to SNVs and SNPs as mutations. We encode the presence or absence of mutations in the cells by an $n \times m$ binary *mutation matrix* *A* where $a_{i,j} = 1$ if cell *i* contains mutation *j* and $a_{i,j} = 0$ indicates the mutation *j* is absent in cell *i*. A phylogenetic tree *T* for the tumor is a rooted node-labeled tree which describes the evolutionary history of the tumor. Each internal node *v* in the tree *T* represents an ancestral cell and is labeled by a vector $a_v\in \{0,1\}^m$ indicating the presence/absence of each mutation $j\in \{1,\ldots ,m\}$ in that cell. The root *r*(*T*) of the tree *T* represents the normal cell. As such $a_{r(T), j} = 0$ if mutation *j* is an SNV, i.e., a somatic mutation, and $a_{r(T), j} = 1$ if mutation *j* is an SNP, i.e., a germline mutation. Each leaf of *T* corresponds to one of the *n* cells in the tumor. Our goal is to reconstruct a phylogenetic tree *T* for a given mutation matrix *A* under a given evolutionary model.

An edge (*v*, *w*) of a phylogeny *T* induces the *gain* of a mutation *j* if $a_{v,j} = 0$ and $a_{w,j} = 1$. On the other hand, a mutation *j* is said to be *lost* on edge (*v*, *w*) if $a_{v,j} = 1$ and $a_{w,j} = 0$. The simplest evolutionary model used in cancer genomics is the *infinite-sites model* which has two constraints [21]. Firstly, a mutation is allowed to be gained at most once in the phylogeny. This constraint stems from the *infinite-sites assumption* which posits that it is very unlikely for the same position in the genome to get mutated multiple times independently. Secondly, once a mutation is gained it cannot be subsequently lost. A phylogeny that satisfies these constraints is known as a perfect phylogeny [40].

While parallel mutations (i.e., the same mutation gained multiple times in the phylogeny) and back mutations (i.e., a mutation reverted back to reference state) are rare in cancer [22], SNVs and SNPs are frequently lost due to copy-number aberrations. As such, more recent phylogeny inference methods [25, 32] apply some variant of the *Dollo model* [28] for phylogeny inference, which allows loss of SNVs/SNPs. Specifically, under the Dollo model, a mutation is allowed to be gained at most once but can be lost multiple times in the phylogeny. The parameterized version of this model is the *k*-Dollo model, in which a mutation can only be lost at most *k* times in the phylogeny. However, a major limitation of Dollo models is that, although they allow loss of SNVs and SNPs, possibly due to CNAs, they do not incorporate any information about the copy-number states of the cells.

We introduce the constrained *k*-Dollo model that supplements the *k*-Dollo model with two additional constraints using the copy-number clustering of the cells. First, since reversal of mutations (*back mutations*) in cancer are rare [22, 23], we assume that SNVs and SNPs can only be lost due to overlapping CNAs. As such, we only allow such losses between cells that belong to distinct copy-number clusters. Second, we assume that each copy-number profile describing the copy number states over the entire genome arises only once in the phylogeny. As such, cells belonging to the same cluster form a connected subtree in the phylogeny. Let *p* be the number of copy-number clones and $\sigma$ be the copy-number clustering of the *n* cells. We formally define the *constrained* *k-Dollo phylogeny* for a mutation matrix *A* and copy-number clustering $\sigma$ as follows.

#### Definition 1

(constrained *k*-Dollo phylogeny) A *constrained* *k-Dollo phylogeny* *T* has the following properties.

1. Each node *v* in *T* is labeled by $a_v\in \{0,1\}^m$ and a copy number clone $\sigma (v)$.
2. The root *r*(*T*) is labeled such that $a_{r(T),j} = 0$ if mutation *j* is an SNV and $a_{r(T), j} = 1$ if *j* is an SNP.
3. For each mutation *j*, there is at most one edge (*v*, *w*) in *T* such that $a_{v,j} = 0$ and $a_{w,j} = 1$.
4. For each mutation *j*, there are at most *k* edges (*v*, *w*) in *T* such that $a_{v,j} = 1$ and $a_{w,j} = 0$.
5. For edge (*v*, *w*) in *T* such that $a_{v,j} = 1$ and $a_{w,j} = 0$ for some mutation *j*, we have $\sigma (v) \ne \sigma (w)$.
6. For any copy number clone $\ell$, the set of nodes labeled $\sigma (v) = \ell$ form a connected subtree of *T*.

We say that a $n\times m$ binary matrix *A* is a constrained *k*-Dollo phylogeny matrix for copy-number clustering $\sigma$ if and only if there exists a constrained *k*-Dollo phylogeny *T* for *A* and $\sigma$, i.e., *T* has *n* leaves and for each leaf there is a *unique* index $i\in \{1,\ldots , n\}$ such that the leaf is labeled by the row $a_i$ of *A* and $\sigma (i)$.

The constrained *k*-Dollo model generalizes the infinite sites model [21] and the *k*-Dollo model [28]. Specifically, when the number *p* of clusters is 1, the constrained *k*-Dollo model is equivalent to the infinite sites model. On the opposite extreme, when the number *p* of clusters is equal to the number *n* of cells, i.e., each cell is in a distinct cluster, the constrained *k*-Dollo model is equivalent to the *k*-Dollo model.

#### Constrained *k*–Dollo phylogeny problem for read count data

During a scDNA-seq experiment, we do not observe the mutation matrix *A* directly. Instead, we observe read counts for each mutation in each cell. Specifically, we obtain the variant read count matrix $Q \in \mathbb {Z}^{n\times m}$, where $q_{i,j}$ is the number of reads with the variant allele for mutation *j* in cell *i*, and the total read count matrix $R \in \mathbb {Z}^{n\times m}$, where $r_{i,j}$ is the total number of reads for mutation *j* in cell *i*. Considering that the cells and mutations in each cell are sequenced independently, and given the tree, the cells evolve independently, the likelihood of observing the variant read count matrix *Q* for given total read count matrix *R* and mutation matrix *A* can be written as follows.

1 $$\begin{aligned} \Pr (Q\mid R, A) = \prod _{i=1}^n\prod _{j=1}^m \Pr (q_{i,j}\mid r_{i,j}, a_{i,j}). \end{aligned}$$

We model the observed variant read counts $q_{i,j}$ using a beta-binomial, similar to previous work [32, 41, 42]. The “Methods” section provides the details about the read count model.

For given read count matrices *Q* and *R*, copy-number clustering $\sigma$ of the cells and integer *k*, our goal is to construct a constrained *k*-Dollo phylogeny that maximizes the likelihood described in Eq. 1. We refer to this as the *Constrained* *k-Dollo phylogeny problem for read count data* and pose it as follows.

##### Problem 1

(Constrained *k*-Dollo phylogeny problem for read count data (C*k*DP-RC)) Given a variant read count matrix *Q*, total read count matrix *R*, copy-number clustering $\sigma$ and integer *k*, find mutation matrix *A* and phylogeny *T* such that (i) likelihood $\Pr (Q\mid R, A)$ is maximized and (ii) *T* is a constrained *k*-Dollo phylogeny for *A* and $\sigma$.

In the “Methods” section, we describe a combinatorial characterization of constrained *k*-Dollo phylogenies that we incorporate in an efficient mixed linear integer program (MILP) to solve the C*k*DP-RC problem. Our resulting method, ConDoR, is implemented in Python 3 using Gurobi [43] (version 9.0.3) to solve the MILP. ConDoR is available at https://github.com/raphael-group/ConDoR.

### Evaluation on simulated data

We compare ConDoR to SCARLET [32], SPhyR [25], SiFit [29] and SCITE [17] on simulated data. We generated simulated data with $n\in \{25, 50, 100\}$ cells, $m\in \{25, 50, 100\}$ mutations, $p\in \{3,5\}$ copy-number clusters, and maximum number of losses $k\in \{1,2,3\}$. We used a growing random network [44] to generate a tree *T* with $m + p$ edges and assign mutations, copy number states, and cluster assignments to each tree, as described in the “Methods” section. Next, we assign *n* cells uniformly at random to one of the nodes in the tree. We simulate the sequencing data for each mutation in each cell using a beta-binomial read count model (details in the “Methods” section). We simulate 5 instances for each combination of the varying simulation parameters. The precise input parameters used for each method are described in Additional file 1: Section C.

We compare the mutation matrix $\hat{A} = [\hat{a}_{i,j}]$ and tumor phylogeny $\hat{T}$ inferred by each method to the ground truth as follows. Following previous studies [25, 32], we evaluate the inferred mutation matrix $\hat{A}$ against the ground-truth mutation matrix *A* by computing the normalized mutation matrix error $\epsilon (A, \hat{A})$ between *A* and $\hat{A}$ given by,

$$\begin{aligned} \epsilon (A, \hat{A}) = \frac{1}{nm} \sum \limits _{i=1}^n\sum \limits _{j=1}^m |a_{i,j} - \hat{a}_{i,j}|. \end{aligned}$$

We evaluate the accuracy of the inferred tumor phylogeny $\hat{T}$ compared to the ground-truth tumor phylogeny *T* by computing the *pairwise ancestral relation accuracy* $E(T, \hat{T})$ [25, 32]. Specifically, under the assumption that a mutation can be gained only once in the phylogeny, which is employed by all methods that we compare against in this study except SiFit, we compute the accuracy of inferring the correct relationship between all possible pairs of mutations from the inferred tumor phylogeny (details in the “Methods” section). When the pairwise ancestral relation accuracy $E(T, \hat{T})$ is 1, the inferred tumor phylogeny $\hat{T}$ and the ground-truth tumor phylogeny *T* are identical when restricted to the edges on which the mutations are gained. We exclude SiFit when computing this metric because it uses the finite-sites model, which allows mutations to occur multiple times in the phylogeny as a consequence of which pairs of mutations may not have a unique relationship.

**Fig. 2:**
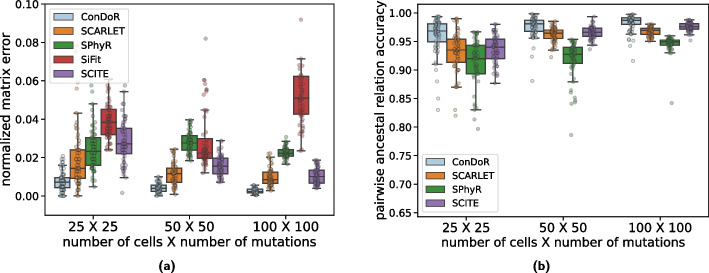
ConDoR outperforms existing methods in recovering the mutation matrix and the tumor phylogeny on simulated data. **a** Normalized mutation matrix error and **b** pairwise ancestral relation accuracy for each method compared to the simulated ground truth. Box plots show the median and the interquartile range (IQR), and the whiskers denote the lowest and highest values within 1.5 times the IQR from the first and third quartiles, respectively

ConDoR outperforms all the other methods in terms of both the normalized mutation matrix error (Fig. 2a) and the ancestral relationship accuracy (Fig. 2b) across all simulation parameters. For instance, on the largest simulated instances with $n = 100$ cells and $m = 100$ mutations, ConDoR achieves the lowest normalized mutation matrix error (median $\epsilon (A,\hat{A}) = 0.002$) and the highest pairwise ancestral relation accuracy (median $E(T,\hat{T}) = 0.986$) compared to SCARLET ($\epsilon (A,\hat{A}) = 0.008$, $E(T,\hat{T}) = 0.969$), SCITE ($\epsilon (A,\hat{A}) = 0.01$, $E(T,\hat{T}) = 0.975$), SiFit ($\epsilon (A,\hat{A}) = 0.05$), and SPhyR ( $\epsilon (A,\hat{A}) = 0.02$, $E(T,\hat{T}) = 0.949$). The superior performance of ConDoR comes with running times comparable to existing methods, although ConDoR does have a higher runtime on some of the large simulated instances with $n=100$ cells and $m=100$ mutations (Additional file 1: Fig. S5).

Interestingly, ConDoR outperforms SCARLET even though SCARLET is given substantially more information about copy number aberrations including both the precise copy-number profile of each cell and the true copy-number tree as input. We believe that this advantage is due to ConDoR solving the underlying optimization problem exactly while SCARLET employs various heuristics that are not guaranteed to yield an optimal solution. Using additional simulations, we also show that ConDoR is robust to noise in the copy number clustering and the read count model parameters (Additional file 1: Section D).

### Multi-region pancreatic ductal adenocarcinoma data

We used ConDoR to analyze targeted single-cell DNA sequencing (scDNA-seq) data from two regions of a pancreatic ductal adenocarcinoma (PDAC) tumor. Specifically, we sequenced two samples (S1 and S2) from distinct regions of the resected tumor using both conventional bulk whole exome sequencing and Mission Bio Tapestri single-cell sequencing (details in the “Methods” section). The scDNA-seq workflow was conducted using a targeted panel consisting of 596 amplicons (median length is 209 bps, Additional file 1: Fig. S6a) interrogating frequently mutated genes in PDAC. We obtained sequencing data from 2153 cells (1167 cells from the first sample and 986 cells from the second sample) with a median coverage of $67\times$ per amplicon per cell.

**Fig. 3:**
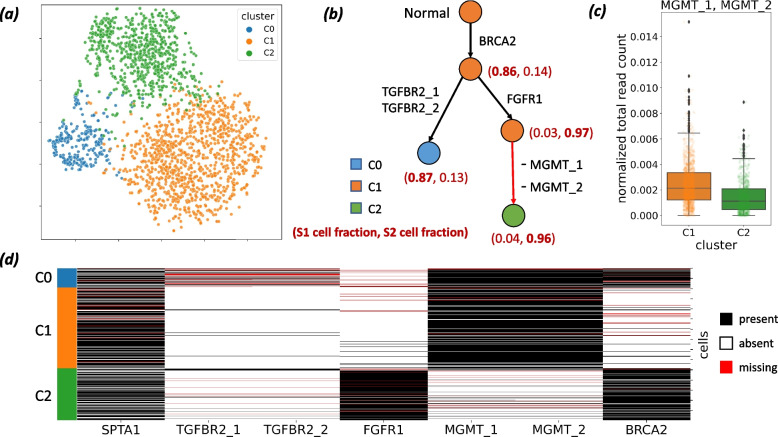
ConDoR provides insights into the evolution and spatial clonal architecture of a pancreatic ductal adenocarcinoma tumor using scDNA-seq data from two different regions of the tumor. **a** *t*-SNE plot showing results of clustering (details in the “Methods” section) of cells into 3 clusters (C0, C1, and C2) according to copy number profiles. **b** Constrained 1-Dollo phylogeny computed by ConDoR with edges labeled by the gain or loss of mutations and vertices labeled by the copy-number cluster and the fraction of cells from samples S1 and S2 that are attached at that vertex. **c** Reduction in normalized total read count for amplicon AMPL257637 (which contains mutations MGMT_1 and MGMT_2) in cells from cluster C2 compared to cells in cluster C1 ($p < 5.8\times 10^{-33}$, a one-sided KS test), supporting the loss of these mutations in the cells belonging to copy-number cluster C2. **d** Observed mutation matrix obtained by discretizing read counts of the 7 mutations, with cells grouped by copy number cluster as indicated in the first column. Box plots show the median and the interquartile range (IQR), and the whiskers denote the lowest and highest values within 1.5 times the IQR from the first and third quartiles, respectively

We identified 7 mutations of interest―including somatic SNVs in *BRCA2*, *TGFBR2*, *FGFR1* and germline SNPs in *SPTA1*, *MGMT*. These mutations were identified using matched bulk tumor and normal sequencing data and were present in the single-cell data with high confidence (details in “Methods” section). Due to the short length of amplicons and uneven distribution in coverage (Additional file 1: Fig. S6b), accurate copy-number calling using this data is challenging. Instead we clustered cells according copy number profiles derived from normalized read counts using *k*-means clustering [45] for number of clusters $p\in \{2,\ldots ,8\}$. We select the best value for *p* using the Silhouette score [46] (see “Methods” section for details). This analysis reveals 3 copy-number clusters (Fig. 3a), which we label C0, C1, and C2, that contain 275, 1145, and 733 cells, respectively.

ConDoR produces a more plausible phylogeny of the PDAC tumor compared to existing methods and provides insights into the evolution of tumor. While most PDAC cases are driven by canonical gain-of-function *KRAS* mutations [47], ConDoR reveals that the tumor analyzed here is driven by a truncal *BRCA2* stop-gained mutation (p.Y600*), which likely inactivated the BRCA2 protein, a tumor suppressor essential for homologous recombination repair [48]. The ConDoR phylogeny shows branched evolution of the tumor, with the trunk leading to two branches (Fig. 3b), the first characterized by two missense *TGFBR2* mutations, TGFBR2_1 (p.A426G) and TGFBR_2 (p.M425I), which likely inactivated cell-intrinsic TGF-$\beta$ signaling, and the second characterized by a missense mutation to *FGFR1* (p.T50K). Although *FGFR1* is involved in MAPK-ERK signaling [47], the particular point mutation’s significance is yet to be characterized. ConDoR infers loss of two germline SNPs in *MGMT*, MGMT_1 and MGMT_2 (both contained in gene MGMT and amplicon AMPL257637), on the edge in the second branch distinguishing cells of cluster C2 from cells of cluster C1. This suggests a loss of heterozygosity (LOH) in cluster C2 cells, which is supported by lower normalized total read count of their amplicon (AMPL257637) in the cells from cluster C2 compared to the cells from cluster C1 (Fig. 3c, $p < 5.8\times 10^{-33}$ with a one-sided Kolmogorov-Smirnov test). Lastly, the root of the ConDoR phylogeny is labeled by cluster C1, indicating that it contains the normal cells present in the data.

We compared the ConDoR phylogeny to the phylogenies produced by two other methods on this data: COMPASS [34], which infers a comprehensive phylogeny with both SNV and CNA events, and SPhyR [25], which uses the *k*-Dollo model. We could not run SCARLET on this data because it was difficult to obtain reliable integer copy numbers and copy number trees from this targeted sequencing data. While COMPASS takes the read count matrices as input, SPhyR takes an observed mutation matrix (Fig. 3d) obtained by discretizing read counts (details in the “Methods” section). COMPASS hypothesizes 8 loss of heterozygosity events (Additional file 1: Fig. S7a) covering all genes in the study except *SPTA1* (*BRCA2*, *TGFBR2*, *FGFR1*, *MGMT*). SPhyR (with $k = 1$) produces a phylogeny that contains loss of all the mutations except *FGFR1* (Additional file 1: Fig. S7b), which is highly unlikely. This demonstrates that using permissive models, like the ones used in COMPASS and SPhyR, may lead to overfitting of the data resulting in overestimation of mutations with loss. ConDoR’s constrained *k*-Dollo model avoids overfitting by incorporating the copy-number clustering to constrain where loss of mutations can occur in the phylogeny.

The phylogeny constructed by ConDoR also reveals a spatial clonal architecture of the PDAC tumor that agrees with previous histological analysis of the tumor. Specifically, the ConDoR phylogeny shows an enrichment of cells from sample S2 (743 cells from S2 vs. 28 cells from S1) in the second branch of the phylogeny, characterized by the edge with the mutation in *FGFR1* (Fig. 3b). Such a spatial separation of the two clonal lineages conforms to histopathological results of this tumor (Fig. 5 in [15]) that showed two populations of tumor cells with distinct morphologies that were well demarcated. Spatial structure in the clonal heterogeneity of tumors has also been observed in previous cancer studies and has several clinical implications such as resistance to therapy and recurrences [49–52]. In summary, ConDoR leverages copy-number clustering obtained from targeted scDNA-seq data to build a more plausible tumor phylogeny compared to existing methods and reveals the spatial structure of the intra-tumor heterogeneity.

### Metastatic colorectal cancer data

We also analyzed a published targeted scDNA-seq dataset from a metastatic colorectal cancer patient CRC2  [37]. This dataset consists of 36 SNVs that were identified from a 1000 gene panel in 186 cells: 145 from the primary tumor and 41 from a liver metastasis. The original study built a phylogeny of the 186 cells using SCITE [17] and reported two distinct branches of metastatic cells on this phylogeny. This phylogeny suggests a polyclonal origin of the metastasis, i.e., the metastatic tumor was seeded by two distinct clones that migrated from the primary tumor (Additional file 1: Fig. S8a). To evaluate the accuracy of the SCITE tree, the authors identified two *bridge mutations*, in the genes FHIT and ATP7B, that were present in the cells of the second metastatic branch (detected in 10/13 and 13/13 cells, respectively) but absent in the cells of the first metastatic branch (detected in 1/15 and 1/15 cells, respectively).

Two subsequent analyses of this data―using the PhISCS [20] and SCARLET [32] algorithms―yield a simpler explanation for the data; namely that the liver metastasis resulted from monoclonal seeding; i.e., the metastatic tumor resulted from a single migration from the primary tumor. However, neither of these studies adequately explain the absence of the bridge mutations in cells of the second metastatic branch in the SCITE tree. PhISCS removed the bridge mutations from analysis in order to obtain a perfect phylogeny that supports monoclonal seeding. SCARLET, using a loss-supported Dollo model, found evidence for the loss of the FHIT mutation due to a deletion in some cells (Additional file 1: Fig. S8b) but concluded that the absence of the ATP7B mutation in all the cells from the second metastatic branch in the SCITE tree was due to simultaneous false negatives in all these cells, a highly unlikely scenario.

**Fig. 4:**
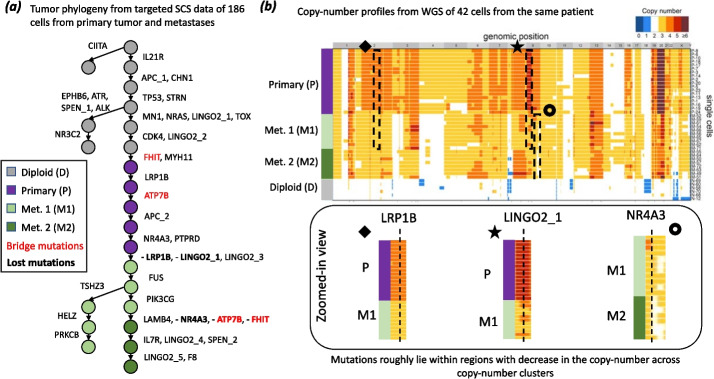
ConDoR infers a phylogeny that is consistent with the copy-number clones in a metastatic colorectal cancer dataset. **a** The ConDoR phylogeny shows loss of bridge mutations FHIT and ATP7B and suggests monoclonal origin of the liver metastasis. **b** Losses inferred by ConDoR are supported by copy-number profiles from whole genome sequencing data of 42 cells from the same patient in the original study [37] (heatmap showing copy-number profiles adapted from [37]). Mutations LRP1B, LINGO2_1, and NR4A3 lie in regions (black boxes) that decrease in copy-number between the clusters that label the vertices on the edge in the phylogeny where the corresponding mutation (bold text in (**a**)) is lost : P$\rightarrow$M1 for LRP1B and LINGO2_1, and M1$\rightarrow$M2 for NR4A3

ConDoR produces a phylogeny that both supports monoclonal seeding of the metastasis *and* provides a more plausible explanation for the absence of the bridge mutations in some of the metastatic cells compared to previous analyses. The ConDoR phylogeny was produced using the copy-number clustering from [32], which included 4 clusters: 120 diploid cells (D), 33 aneuploid profile of primary tumor cells (P), and two distinct aneuploid profiles of metastatic tumor cells (M1 and M2 with 23 and 10 cells, respectively). The ConDoR tree contains a single branch containing all the metastatic cells, supporting the simpler hypothesis of monoclonal seeding of the liver metastasis, in agreement with the PhISCS and SCARLET analyses (Fig. 4a). Moreover, ConDoR infers the loss of both the bridge mutations, FHIT and ATP7B, leading to a phylogeny with a higher likelihood compared to SCARLET (log-likelihood − 8324.8 for ConDoR and − 8437.4 for SCARLET). This demonstrates that the low resolution of the copy-number aberrations derived from targeted scDNA-seq data used by SCARLET may lead to misleading results, and ConDoR avoids these errors by only using the copy-number clusters.

We also find that the ConDoR tree is consistent with copy-number profiles obtained from whole-genome sequencing of 42 additional cells from the same patient. These cells were not used in the phylogenetic analyses. In addition to the bridge mutations, ConDoR infers the loss of SNVs in LRP1B, LINGO2_1, and NR4A3. These three SNVs lie within regions with lower copy numbers in the WGS copy-number profiles from the original study (Fig. 4b). The copy-number profiles from WGS data also reveal that all metastatic cells share copy-number deletions in chromosomes 2, 3p, 4, 7, 9, 16, and 22 relative to the cells in the primary tumor. These shared copy number profiles further corroborates the ConDoR tree (and the PhISCS and SCARLET trees) in which all metastatic cells are in a single clade. In contrast, SCITE tree from the original study suggests that these CNAs occurred independently in the two distinct branches of the phylogeny with metastatic cells which is a less likely explanation. In summary, ConDoR integrates SNVs and copy-number clustering to build a tumor phylogeny that contains loss of SNVs that are supported by orthogonal copy-number data and supports a simpler monoclonal origin of the metastasis compared to the original study.

## Conclusions

We introduced a new evolutionary model, the constrained *k*-Dollo model, a model for two-state phylogenetic characters, in which a character can be gained at most once and lost at most *k* times but where the losses are constrained according to a given clustering of the taxa. This model was inspired by the challenge of inferring a phylogenetic tree from targeted single-cell DNA sequencing data, where SNVs and SNPs are measured with high fidelity, but CNAs are poorly described. Specifically, our model relies on a clustering of cells based on their copy-number profiles as input, without requiring identification of precise CNAs in each cell. The constrained *k*-Dollo model generalizes both the infinite sites model and the *k*-Dollo model.

We developed an algorithm, ConDoR (Constrained Dollo Reconstruction), that infers the most parsimonious constrained *k*-Dollo phylogeny using a probabilistic model for the read counts in scDNA-seq data. On simulated data, ConDoR outperforms state-of-the-art tumor phylogeny inference methods. On a multi-region targeted scDNA-seq data of pancreatic ductal adenocarcinoma tumor, ConDoR produced a more plausible phylogeny compared to existing methods, providing insights into the evolution and spatial clonal architecture of the tumor. On targeted scDNA-seq data of metastatic colorectal cancer patient, ConDoR found a phylogeny that supports a simpler monoclonal origin of liver metastasis compared to polyclonal seeding proposed by the original study [37].

There are several limitations and directions for future research. First, ConDoR currently takes the read count matrices with the mutations and the copy-number clustering as input to build a constrained *k*-Dollo phylogeny. A future direction is to extend ConDoR to simultaneously perform variant calling, and inference of the copy-number clustering and the phylogeny, potentially improving the accuracy of the results. Second, ConDoR and several existing methods [17, 20, 25, 29, 32] disregard the location of SNVs during phylogeny inference. However, since CNAs alter the copy-number of contiguous segments of the genome, the SNV locations can be used to model the likelihood of simultaneous loss of multiple adjacent SNVs. Lastly, while ConDoR only uses scDNA-seq data as input, the underlying constrained *k*-Dollo model is a general model for evolution of SNVs. We propose that this model can be used for phylogeny inference while integrating information from multiple sequencing technologies, possibly measuring different modalities of the cancer cells [19, 53].

## Methods

### Characterization of constrained *k*-Dollo phylogenies

We derive a characterization of constrained *k*-Dollo phylogeny matrices by building on previous work on characterization of *k*-Dollo phylogeny matrices [25, 26]. Recall that in the *k*-Dollo model, a 0 entry in the mutation matrix *A* indicates that either the mutation did not occur in the cell or that the mutation occurred but then was subsequently lost in the cell. If we could distinguish these two cases, then we could replace the 0 entries resulting from losses by additional character states $\{2,\ldots ,k+1\}$ representing the *k* possible losses of a mutation in the *k*-Dollo phylogeny. This idea forms the basis of the following (extended) definition of *k-completion of a mutation matrix* *A*.

#### Definition 2

(El-Kebir 2018 [25]) A matrix $B\in \{0,\ldots , k+1\}^{n\times m}$ is a *k* ***-completion*** of a mutation matrix $A\in \{0,1\}^{n\times m}$ provided: (1) $b_{i,j} = 1$ if and only if $a_{i,j} = 1$; (2) $b_{i,j}\in \{0,\ldots , k+1\}\setminus \{1\}$ if and only if $a_{i,j} = 0$; (3) $b_{i,j} \ge 1$ if *j* is an SNP.

The following definition defines a subset of all possible *k*-completion matrices of a mutation matrix *A*.

#### Definition 3

(El-Kebir 2018 [25]) A matrix $B\in \{0,\ldots ,k+1\}^{n\times m}$ is a *k* ***-Dollo completion*** of mutation matrix *A* provided it is a *k*-completion of mutation matrix *A* such that there exists no two columns and three rows in *B* of the following forms:

$$\begin{aligned} \left( \begin{array}{cc} i_1 &{} 0\\ 0 &{} j_1 \\ i^{\prime }_1 &{} j^{\prime }_1 \end{array}\right) \text { or }&\left( \begin{array}{cc} i_1 &{} j_1^{\prime \prime }\\ 0 &{} j_2 \\ i^{\prime }_1 &{} j_2 \end{array}\right) \text { or } \left( \begin{array}{cc} i_2 &{} 0\\ i^{\prime \prime }_1 &{} j_1 \\ i_2 &{} j^{\prime }_1 \end{array}\right) \text { or } \\&\left( \begin{array}{cc} i_2 &{} j_1^{\prime \prime }\\ i^{\prime \prime }_1 &{} j_2 \\ i_2 &{} j_2 \end{array}\right) , \end{aligned}$$

where $i_1,i'_1,j_1,j'_1\in I^{(1)}, i_2,j_2\in I^{(2)}, i^{\prime \prime }_1\in I^{(1)}\setminus \{i_2\}$ and $j^{\prime \prime }_1\in I^{(1)}\setminus \{j_2\}$, and $I^{(i)} = \{i,\ldots , k+1\}$.

According to this definition, the number of $3\times 2$ submatrices that are forbidden to exist in *k*-Dollo completion matrices is $(k+1)^4 + 2k^2(k+1)^2 + k^4$. Ciccolella et al.[26] provided an alternate characterization of *k*-Dollo completion matrices, which we describe in Additional file 1: Section B.

*k*-Dollo completion matrices are useful in characterization of *k*-Dollo phylogeny matrices due to the following theorem.

#### Theorem 1

(El-Kebir 2018 [25]) $A\in \{0,1\}^{n\times m}$ is a *k*-Dollo phylogeny matrix if and only if there exists a *k*-Dollo completion $B\in \{0,\ldots ,k+1\}^{n\times m}$ of *A*.

Constrained *k*-Dollo phylogenies are a subset of *k*-Dollo phylogenies that satisfy some additional constraints. In particular, a constrained *k*-Dollo completion must be *consistent* with copy-number clustering $\sigma$, according to the following definition.

#### Definition 4

(Consistency) A *k*-Dollo completion $B\in \{0,\ldots ,k+1\}^{n\times m}$ of a mutation matrix *A* is *consistent* with a copy-number clustering $\sigma$ with *p* clusters provided the following conditions are true for every mutation *j*.

1. There is at most one cluster $\ell$ such that for two distinct cells $i, i' \in \sigma ^{-1}(\ell )$, $b_{i,j} = 0$ and $b_{i',j} = 1$.
2. If there exists cell *i* such that $\sigma (i) = \ell$ and $b_{i,j} = s$ for $s\in \{2,\ldots , k+1\}$, then $b_{i',j} = s$ for all $i'\in \sigma ^{-1}(\ell )$.

Using this definition, we have the following characterization of constrained *k*-Dollo phylogeny matrices.

#### Theorem 2

A mutation matrix *A* is a constrained *k*-Dollo phylogeny matrix for copy-number clustering $\sigma$ if and only if there exists a *k*-Dollo completion $B\in \{0,\ldots , k+1\}^{n\times m}$ of *A* that is consistent with $\sigma$.

We provide a proof of Theorem 2 in Additional file 1: Section A and show that given a *k*-Dollo completion *B* of mutation matrix *A* that is consistent with $\sigma$, we can find a constrained *k*-Dollo phylogeny for *A* and $\sigma$ in *O*(*nmk*) time. In addition, we also show the following result on the complexity of the C*k*DP-RC problem (Problem 1).

#### Theorem 3

The C*k*DP-RC problem is NP-hard, even for $k = 0$.

A proof of Theorem 3 is provided in Additional file 1: Section A.

### ConDoR algorithm for constrained *k*-Dollo model

We formulate a mixed integer linear program (MILP) to solve Problem 1 exactly. Specifically, for given read count matrices *Q* and *R*, copy-number clustering $\sigma$ and integer *k*, the MILP finds a *k*-Dollo completion *B* that is consistent with $\sigma$ and that maximizes the likelihood $\Pr (Q\mid R, A)$, where *A* is the mutation matrix corresponding to *B*.

The MILP is based on encoding the combinatorial characterization of constrained *k*-Dollo completion matrices described in the previous section. We introduce a binary variables $a_{i,j}$ for cell *i* and mutation *j* to represent the mutation matrix *A*. Further, we introduce binary variables $c_{\ell ,j,s}$ for cluster $\ell$, mutation *j* and state $s\in \{2,\ldots ,k+1\}$ to represent the presence of loss state *s* of mutation *j* in cluster $\ell$. These binary variables are used to model the entries of the *k*-completion matrix *B* as follows: $b_{i,j} = 1$ if $a_{i,j} = 1$; $b_{i,j} = s$ if $c_{\ell ,j,s} = 1$ and $\sigma (i) = \ell$ for $s\in \{2,\ldots ,k+1\}$; and $b_{i,j} = 0$ otherwise.

Since $b_{i,j}$ can only attain one value, we enforce the following constraints for all cells $i\in \{1,\ldots , n\}$ and all mutations $j\in \{1,\ldots , m\}$.

$$\begin{aligned} a_{i,j} + \sum \limits _{s=2}^{k+1} c_{\sigma (i),j,s} \le 1. \end{aligned}$$

We also define variables $x_{i,j}$ for cell *i* and mutation *j* which indicates if $b_{i,j} \ge 1$. As such, for all cells $i\in \{1,\ldots , n\}$ and all mutations $j\in \{1,\ldots , m\}$ we enforce

$$\begin{aligned} x_{i,j} = a_{i,j} + \sum \limits _{s=2}^{k+1} c_{\sigma (i),j,s}. \end{aligned}$$

Once we have modeled the *k*-completion matrix *B*, we need to enforce constraints for (i) consistency with the copy-number clustering $\sigma$, (ii) handling germline mutations and (iii) *B* to be a *k*-Dollo completion matrix. We describe the constraints for (i), (ii) and the objective function of the MILP in the following and refer to Additional file 1: Section B for (iii).

#### Handling germline mutations

 Here, we describe the constraints to handle germline mutations. Note that, if mutation *j* is germline, it must either be present in cell *i*, i.e., $a_{i,j} = 1$, or it must have been lost, i.e., $c_{\sigma (i),j,s} = 1$ for some $s\in \{2,\ldots ,k+1\}$. As such, if $G\subseteq \{1,\ldots , m\}$ is the set of germline mutations, we enforce the following constraints for all cells $i\in \{1,\ldots , n\}$ and germline mutations $j\in G$,

$$\begin{aligned} a_{i,j} + \sum \limits _{s=2}^{k+1} c_{\sigma (i),j,s} = 1. \end{aligned}$$

#### Consistency constraints

 We now describe the constraints to enforce consistency between the *k*-completion matrix *B* and the copy-number clustering $\sigma$. Note that Condition 2 of Definition 4 is satisfied by the way *B* is modeled, and we only need to introduce constraints to satisfy Condition 1 of Definition 4. To that end, we introduce two set of continuous auxiliary variables. First, we introduce $g^{(0)}_{\ell ,j}\in [0,1]$ and enforce constraints so that $g^{(0)}_{\ell ,j} = 1$ if there exists at least one cell $i\in \sigma ^{-1}(\ell )$ such that $b_{i,j} = 0$ for cluster $\ell$ and mutation *j*, and $g^{(0)}_{\ell ,j} = 0$ otherwise. Similarly, we introduce $g^{(1)}_{\ell ,j}\in [0,1]$ and enforce constraints so that $g^{(1)}_{\ell ,j} = 1$ if there exists at least one cell $i\in \sigma ^{-1}(\ell )$ such that $b_{i,j} = 1$ for cluster $\ell$ and mutation *j*, and $g^{(1)}_{\ell ,j} = 0$ otherwise. We model these variables using the following constraints for all mutations $j\in \{1,\ldots , m\}$ and clusters $\ell \in \{1,\ldots , p\}$,

$$\begin{aligned} \begin{array}{lr} g^{(0)}_{\ell ,j} \ge 1 - x_{i,j},\quad &{}\text {for all}\ i\in \sigma ^{-1}(\ell ),\\ g^{(0)}_{\ell ,j} \le |\sigma ^{-1}(\ell )| - \sum \limits _{i\in \sigma ^{-1}(\ell )} x_{i,j},\quad &{}\\ g^{(1)}_{\ell ,j} \ge a_{i,j},\quad &{}\text {for all}\ i\in \sigma ^{-1}(\ell ),\\ g^{(1)}_{\ell ,j} \le \sum \limits _{i\in \sigma ^{-1}(\ell )} a_{i,j}.\quad &{} \end{array} \end{aligned}$$

Next, we introduce continuous variables $g_{\ell , j}\in [0,1]$ such that $g_{\ell ,j} = 1$ if and only if mutation *j* is gained in cluster $\ell$ and $g_{\ell ,j} = 0$ otherwise, for cluster $\ell$ and mutation *j*. Specifically, $g_{\ell ,j} = 1$ if there exists two distinct cells $i, i'\in \sigma ^{-1}(\ell )$ such that $b_{i,j} = 0$ and $b_{i',j} = 1$. We use $g^{(0)}_{\ell ,j}$ and $g^{(1)}_{\ell ,j}$ to model $g_{\ell ,j}$ for all mutations $j\in \{1,\ldots , m\}$ and clusters $\ell \in \{1,\ldots , p\}$ with the constraints,

$$\begin{aligned} g_{\ell ,j}\le & {} g^{(0)}_{\ell ,j},\\ g_{\ell ,j}\le & {} g^{(1)}_{\ell ,j},\\ g_{\ell ,j}\ge & {} g^{(0)}_{\ell ,j} + g^{(1)}_{\ell ,j} - 1. \end{aligned}$$

Finally to enforce that each mutation can be gained in at most one cluster, we have the following constraint for all mutations $j\in \{1,\ldots , m\}$,

$$\begin{aligned} \sum \limits _{\ell =1}^p g_{\ell ,j} \le 1. \end{aligned}$$

#### Objective function

Recall that we want to maximize the likelihood function $P(Q\mid R, A)$ (Eq. 1), where *A* is the mutation matrix and *B* is its *k*-Dollo completion consistent with copy-number clustering $\sigma$. After taking $\log$ on both sides in Eq. 1, we can linearize the log-likelihood to get the following objective function.

$$\begin{aligned} \max \sum \limits _{i=1}^n\sum \limits _{j=1}^m\ {}&\Bigl (a_{i,j}\log \Pr (q_{i,j}\mid r_{i,j}, a_{i,j} = 1) +\\&(1 - a_{i,j})\log \Pr (q_{i,j}\mid r_{i,j}, a_{i,j} = 0)\Bigr ). \end{aligned}$$

This MILP has $O(nm + pmk)$ binary variables, $O(m^2k^2 + pm)$ continuous variables, and $O(nm^2k^2)$ constraints.

### Simulation details

In this section, we provide details about the simulations and the input files generated for each method.

#### Simulation of the phylogeny

We used a growing random network [44] to generate a tree *T* with $m + p$ edges. Specifically, starting from the root vertex, *T* is built by iteratively adding child nodes while choosing the parent uniformly at random from the nodes in the tree in that iteration. The root node *r*(*T*) represents the normal cell and is assigned to cluster $\ell = 0$. The edges are then labeled by either the gain of a mutation $j\in \{1,\ldots , m\}$ or change to cluster $\ell \in \{1,\ldots , p\}$. For each edge $(v,w)\in E(T)$ labeled with a change in cluster, we allow loss of the mutations gained along the path from the root *r*(*T*) to node *v* with probability $\lambda = 0.8$. We generate a copy-number profile for each node in the tree, as described below.

#### Simulation of copy-number states

The SCARLET [32] algorithm requires the copy-number profile of each cell, as well as the copy-number tree as input. We simulate the copy-number tree as follows.

Each node of the tree is labeled by a copy-number between 0 and 8 for each mutation *j*. We first initialize the root of the tree with a copy-number profile in which the copy-number for each position is picked uniformly at random between 0 and 8. We then label the remaining nodes as we traverse the tree in a breadth-first order. If the edge $(\pi (w), w)$ does not contain loss of mutation *j*, the copy-number for node *w* is the same as the copy-number of $\pi (w)$. On the other hand, if the edge $(\pi (w), w)$ induces the loss of mutation *j*, the copy-number at *j* for node $\pi (w)$ is picked uniformly at random between 1 and 8, while the copy-number of node *w* is picked uniformly at random between 0 and $\pi (w)-1$. This ensures that, (i) the copy-number profile only changes if there is a loss event on the edge and (ii) each loss of mutation is supported by decrement of copy-number. Let $C\in \{0,\ldots ,8\}^{n\times m}$ be the copy-number matrix such that $c_{i,j}$ is the copy-number at locus *j* in cell *i*. This copy-number matrix is used during simulation of the variant read counts which we describe in the next subsection.

#### Read count model

The total read count $r_{i,j}$ for each cell *i* and mutation *j* is modeled as Poisson variable with mean coverage $\text {cov} = 50$, i.e.

$$\begin{aligned} r_{i,j}\sim \text {Poi}(\text {cov}). \end{aligned}$$

We use beta-binomial model, similar to previous works [32, 41, 42] for the variant read count $q_{i,j}$ for each cell *i* and mutation *j*. Our model accounts for sequencing errors and allelic imbalance during sequencing as follows. For sequencing error, we set error rate $\epsilon = 0.001$ which is similar to the error rates of most recent Illumina sequencing platforms [54]. Specifically, we assume that the false positive rate $\alpha _{fp}$ and the false negative rate $\alpha _{fn}$ of observing a read with the variant allele is $\epsilon$. When the mutation *j* is not present in cell *i*, i.e., $a_{i,j} = 0$, the number of copies of the variant allele is 0. When the mutation *j* is present in cell *i*, i.e., $a_{i,j} = 1$, we assume that the number of copies of the variant allele is 1. As such, the value of $a_{i,j}$ indicates the number of variant allele. Given that the total copies of the locus for mutation *j* in cell *i* is $c_{i,j}$, the true variant allele frequency, which we denote by $y_{i,j}$, is given by $y_{i,j} = a_{i,j} / c_{i,j}$. Due to sequencing errors $\alpha _{fp}$ and $\epsilon _{fn}$, the probability $p_{i,j}$ of producing a read containing the variant allele for mutation *j* in cell *i* is

$$\begin{aligned} p_{i,j} = \left( 1 - \alpha _{fn}\right) \frac{a_{i,j}}{c_{i,j}} + \left( 1 - \frac{a_{i,j}}{c_{i,j}}\right) \alpha _{fp}. \end{aligned}$$

The number of variant reads $q_{i,j}$ is given by

$$\begin{aligned} \pi _{i,j}\sim & {} \text {beta}(p_{i,j}, s),\\ q_{i,j}\sim & {} \text {Binom}(r_{i,j}, \pi _{i,j}), \end{aligned}$$

where, we set the dispersion parameter $s = 15$ in our simulations to simulate allelic imbalance. Finally, we spike-in missing entries in the variant read count matrix *Q* and total read count matrix *R* by setting $q_{i,j} = 0$ and $r_{i,j} = 0$ in $\lfloor d n m \rfloor$ entries where *d* is the rate of missing entries in the data.

While ConDoR and SCARLET take the variant and total read counts as input, several methods (such as SPhyR, SCITE and SiFit) require an observed mutation matrix $A'$ as input. In the following section, we show how we obtained the observed mutation matrix from the simulated read counts.

#### Obtaining the observed mutation matrix from the read counts

Methods such as SPhyR, SCITE, and SiFit take an observed mutation matrix ${A}'\in \{0,1,-1\}^{n\times m}$ as input. This observed mutation matrix ${A}'$ may contain missing entries (represented by $-1$) and errors (false positives and false negatives). The aforementioned methods estimate the true binary mutation matrix *A* and build a tumor phylogeny while correcting the errors and imputing the missing entries in the observed mutation matrix $A'$. We denote the estimated mutation matrix by $\hat{A}$.

We obtain ${A}'$ from the read count matrices *Q* and *R* as follows. We use three filtering parameters to discretize the read count matrices: (i) total read count threshold $r_t = 10$, (ii) variant read count threshold $q_t = 5$, and (iii) variant allele frequency threshold $y_t = 0.1$. We say that mutation *j* is present in cell *i* if and only if the total read count $r_{i,j}$ is greater than or equal to $r_t$, the variant read count $q_{i,j}$ is greater than or equal to $q_t$, and the observed variant allele frequency ${y}'_{i,j} = q_{i,j} / r_{i,j}$ is greater than or equal to $y_t$. Specifically, we set ${a}'_{i,j} = 1$ if $(r_{i,j} \ge r_t) \wedge (q_{i,j} \ge q_t) \wedge ({y}'_{i,j} \ge y_t)$. For the remaining entries, we set ${a}'_{i,j} = 0$ if $r_{i,j} \ge 0$, indicating absence of mutation, and ${a}'_{i,j} = -1$, indicating missing entry, otherwise. The median false positive and false negative rates of the observed mutation matrices of the simulated instances are 0.0018 and 0.158, respectively (Additional file 1: Fig. S1). It is possible that using additional procedures to define a higher quality set of mutations could improve the performance of methods such as SPhyR, SCITE and SiFit.

#### Computation of pairwise ancestral relation accuracy

Here, we describe the computation of *pairwise ancestral relation accuracy* $E(T, \hat{T})$ for two tumor phylogenies *T* and $\hat{T}$. Under the assumption that a mutation can be gained only once in the phylogeny, any pair $(j, j')$ of mutations can be related in exactly one of the following four ways.

1. Mutation *j* occurs along the path from the root to source node of edge on which mutation $j'$ occurs.
2. Mutation $j'$ occurs along the path from the root to source node of edge on which mutation *j* occurs.
3. Mutation *j* and $j'$ occur on the same edge of the phylogeny.
4. Mutation *j* and $j'$ occur on distinct branches of the phylogeny.

We compute the accuracy of inferring the correct relationship between all possible pairs of mutations from the inferred tumor phylogeny.

### Generation and pre-processing of the PDAC data

Here, we provide details regarding the generation and pre-processing of targeted sequencing data of pancreatic ductal adenocarcinoma tumor used in this study.

#### Bulk WES library preparation, sequencing, and variant calling

Genomic DNA was extracted from each tissue using the phenol-chloroform extraction protocol [55] or the QIAamp DNA Mini Kits (Qiagen) [56]. WES library preparation and sequencing were performed by the Integrated Genomics Operation at Memorial Sloan Kettering Cancer (MSKCC, NY). Briefly, an Illumina HiSeq 2000, HiSeq 2500, HiSeq 4000, or NovaSeq 6000 platform was used to target sequencing coverages of $>250\times$ for WES samples.

The raw FASTQ files were processed with the standard pipeline of the Bioinformatics Core at MSKCC. Sequencing reads were analyzed in silico to assess quality, coverage, and aligned to the human reference genome (hg19) using BWA [57]. After read de-duplication, base quality recalibration and multiple sequence realignment were completed with the PICARD Suite [58] and GATK v.3.1 [59]; somatic single-nucleotide variants and insertion-deletion mutations were detected using Mutect v.1.1.6 [60] and HaplotypeCaller v.2.4 [61]. This pipeline generated a set of mutations for every single sample. Then, all mutations of all samples of the same sequencing cohort were pooled as a single set. Each sample’s BAM file was used to compute “fillout” values (total depth, reference allele read counts, alternative allele read counts) for each mutation in the pooled list. Mutation with alternate read count less than 2 across all samples were removed to trim down false positives. The purpose was to rescue mutations that were detected with high confidence in one sample but with low confidence in another sample of the same patient/tumor. This generated the final output in mutational annotation format (MAF).

#### Single-cell DNA sequencing (Tapestri) library preparation and variant calling

Single nuclei were extracted from snap frozen primary patient samples embedded in optimal cutting temperature (OCT) compound using the protocol developed by Zhang et al. [15].

Nuclei were suspended in Mission Bio cell buffer at a maximum concentration of 4000 nuclei/$\mu$l, encapsulated in Tapestri microfluidics cartridge, lysed and barcoded. Barcoded samples were then put through targeted PCR amplification with a custom 596-amplicon panel covering important PDAC mutational hotspots in our sample cohort (table with all the amplicons is available at https://github.com/raphael-group/ConDoR).

The 596-amplicon panel was designed based on curation of bulk whole exome/genome sequencing data of PDAC samples collected by the Iacobuzio lab. The goal was to cover as many PDAC-related SNVs within our patient cohort as possible within a 600-amplicon limit, which was deemed economically optimal. The genes/SNVs of interest were determined by querying several resources, such as cBioportal [62, 63] and openCRAVAT [64]. Particular interest was paid to genes in the *TGF*$\beta$ pathway as relevant mutations are currently being investigated as clinical biomarkers [65]. In addition to the SNVs, we added amplicons to cover as much exon region as possible for genes that are of particular interest for CNV analyses in PDAC: *KRAS*, *TP53*, *SMAD4*, *CDKN2A*, *TGFBR1*, *TGFBR2*, *ACVR1B*, *ACVR2A*, *BMPR1A*, *BMPR1B*, *SMAD2*, *SMAD3*, *MYC*, *GATA6*, *BAP1*, *MUS81*, and *KAT5*.

PCR products were removed from individual droplets, purified with Ampure XP beads and used as templates for PCR to incorporate Illumina i5/i7 indices. PCR products were purified again, quantified with an Agilent Bioanlyzer for quality control, and sequenced on an Illumina NovaSeq. The minimum total read depth was determined by same formula as used in [15].

As described in [15], FASTQ files for single-nuclei DNA libraries were processed through Mission Bio’s Tapestri pipeline with default parameters to arrive at the output H5 file, which mainly consists of two matrices: a cell-by-per-amplicon-read-count matrix $\mathbf {X_1}$, and a cell-by-SNV matrix $\mathbf {X_2}$. Briefly, the pipeline has the following steps.

1. Adaptor sequences are trimmed and align the reads to the hg19 genome (UCSC).
2. The reads are assigned to individual cell barcodes, while filtering out the low-quality cell barcodes. For each of these barcodes, the number of forward reads aligned to each amplicon is used to form matrix $\mathbf {X_1}$.
3. Variant calling is performed to generate a gVCF (genomic variant call format) file from the BAM file for each cell.
4. The cells are jointly genotyped to form a cell-by-SNV matrix $\mathbf {X_2}$.

A more detailed documentation of the pipeline is available at: https://support.missionbio.com/hc/en-us/categories/360002512933-Tapestri-Pipeline. In respect of Mission Bio’s request, the pipeline code is not to be publicized because it contains proprietary information per industry standard. However, the pipeline used in the paper that demonstrated this scDNA-seq library preparation technology [66] is publicly available as a Github repository at https://github.com/AbateLab/DAb-seq. Although we have not formally tested that it performs identically as the Mission Bio pipeline, we believe it is sufficient to replicate our results.

#### Variant calling

We detect 40 mutations in the bulk tumor sample with a variant allele frequency (VAF) of at least 0.05. Out of these 40 mutations, 34 mutations were also detected in the matched normal sample indicating that they were germline mutations. From the remaining 6 somatic mutations, we filter out mutations with low prevalence in the scDNA-seq data. Specifically, we only include mutations with variant allele frequency more than 0.1, read depth of more than 20 and variant read depth of more than 10 in at least $5\%$ of the cells. We end up with 4 somatic mutations: chr3:30715617:C/G (TGFBR2_1), chr3:30715619:G/T (TGFBR2_2), chr8:38314915:G/T (FGFR1), and chr13:32907415:T/A (BRCA2).

Most phylogeny inference methods only consider somatic SNVs as input, and filter out all germline SNPs. However, germline SNPs that have undergone loss in a subset of cells are informative during phylogeny inference. We identify germline SNPs with putative loss by including SNPs with variant allele frequency less than 0.1, variant read depth more than 10, and total read depth less than 20 in at least $15\%$ of the cells. We find 3 such SNPs: chr10:131506283:C/T (MGMT_1), chr10:131506192:C/T (MGMT_2), and chr1:158612236:A/G (SPTA1). In summary, we consider 3 germline SNPs and 4 somatic SNVs in our analysis.

#### Copy-number clustering

In this section, we describe the method to cluster the PDAC cells based on the total reads in each cell. Let $\mathcal {A}$ be the set of amplicons, $\mathcal {G}$ be the set of genes and $\mathcal {A}(g)$ denote the set of amplicons contained in gene $g\in \mathcal {G}$. Let $R^{A}_{i,a} = [r^{A}_{i,a}]$ be the $n\times |\mathcal {A}|$ *read depth matrix* that contains the number of reads in cell *i* and amplicon *a*. We start by normalizing the amplicon-level read depth matrix by the total reads in each cell. Specifically, we form matrix $\bar{R}^{A}\in \mathbb {Z}^{n\times |\mathcal {A}|}$ such that,

$$\begin{aligned} \bar{r}^{A}_{i,a} = \frac{{r}^{A}_{i,a}}{\sum \nolimits _{a\in \mathcal {A}} r^A_{i,a}}. \end{aligned}$$

Next, we assume that all loci in the same gene have the same copy-number. As such, we compute the average normalized read depth as follows

$$\begin{aligned} \bar{r}^{G}_{i,g} = \frac{\sum \nolimits _{a\in \mathcal {A}(g)} \bar{r}^A_{i,a}}{|\mathcal {A}(g)|}. \end{aligned}$$

This step helps nullify some noise in the amplicon-level total read count data. We focus on 30 genes with the highest number of amplicons and perform k-means clustering on the resulting matrix $\bar{R}^{G}$.

We use the Silhouette score to determine the number of copy-number clusters. Additional file 1: Fig. S9a shows the the Silhouette score for increasing number of clusters in the k-means clustering. We choose the clustering with the highest Silhouette score resulting in $p = 3$ clusters as the copy-number clustering $\sigma$. Additional file 1: Fig. S9b shows a t-SNE [67] where each point is a cell labeled by the cluster index from the copy-number clustering $\sigma$.

### Supplementary information

**Additional file 1.** Figures, tables and text describing additional information such as proofs of theorems or additional results.

**Additional file 2.** Peer review history.

## Data Availability

ConDoR software, simulations, and processed real data are publicly available at https://github.com/raphael-group/ConDoR [68] under the MIT license. The results reported in the paper are fully reproducible with the data available in the Github repository. The original CRC data is available on NCBI Sequence Read Archive (https://www.ncbi.nlm.nih.gov/sra) under accession number SRP074289 [69]. The raw FASTQ files of PDAC data analyzed in the present study are not publicly available because they are part of a larger cohort to be published in the near future but are available from the corresponding author on reasonable request. Specifically, this data will be deposited in EGA along with a bigger cohort shortly after submission of our next publication. They will be available under restricted access, as required by the MSKCC Medical Donation Program Data Access Agreement (MSKCC MDP DAA). Readers interested in gaining access through EGA need to contact the data access committee (DAC) of this dataset and start an application. The DAC will try to respond within 2 weeks but may take longer in special conditions. The MSKCC MDP DAA, to be provided by the DAC and include guidelines and restrictions on data usage, must be signed. Once the application is approved, an EGA account will be provided for data access. The time length of access to the data will be determined by the DAC on a case-by-case basis. Further information about EGA can be found at https://ega-archive.org and “The European Genomephenome Archive of human data consented for biomedical research” (https://www.nature.com/ng/journal/v47/n7/full/ng.3312.html).

## References

[1] Tabassum DP, Polyak K (2015). Tumorigenesis: it takes a village. Nat Rev Cancer..

[2] Schwartz R, Schäffer AA (2017). The evolution of tumour phylogenetics: principles and practice. Nat Rev Genet..

[3] Amirouchene-Angelozzi N, Swanton C, Bardelli A (2017). Tumor Evolution as a Therapeutic Target The Impact of Tumor Evolution in Precision Medicine. Cancer Discov..

[4] Fittall MW, Van Loo P (2019). Translating insights into tumor evolution to clinical practice: promises and challenges. Genome Med..

[5] Jiao W, Vembu S, Deshwar AG, Stein L, Morris Q (2014). Inferring clonal evolution of tumors from single nucleotide somatic mutations. BMC Bioinforma..

[6] Popic V, Salari R, Hajirasouliha I, Kashef-Haghighi D, West RB, Batzoglou S (2015). Fast and scalable inference of multi-sample cancer lineages. Genome Biol..

[7] Malikic S, McPherson AW, Donmez N, Sahinalp CS (2015). Clonality inference in multiple tumor samples using phylogeny. Bioinformatics..

[8] El-Kebir M, Oesper L, Acheson-Field H, Raphael BJ (2015). Reconstruction of clonal trees and tumor composition from multi-sample sequencing data. Bioinformatics..

[9] Deshwar AG, Vembu S, Yung CK, Jang GH, Stein L, Morris Q (2015). PhyloWGS: reconstructing subclonal composition and evolution from whole-genome sequencing of tumors. Genome Biol..

[10] El-Kebir M, Satas G, Oesper L, Raphael BJ (2016). Inferring the mutational history of a tumor using multi-state perfect phylogeny mixtures. Cell Syst..

[11] Eaton J, Wang J, Schwartz R (2018). Deconvolution and phylogeny inference of structural variations in tumor genomic samples. Bioinformatics..

[12] Laks E, McPherson A, Zahn H, Lai D, Steif A, Brimhall J (2019). Clonal decomposition and DNA replication states defined by scaled single-cell genome sequencing. Cell..

[13] Morita K, Wang F, Jahn K, Hu T, Tanaka T, Sasaki Y (2020). Clonal evolution of acute myeloid leukemia revealed by high-throughput single-cell genomics. Nat Commun..

[14] Minussi DC, Nicholson MD, Ye H, Davis A, Wang K, Baker T (2021). Breast tumours maintain a reservoir of subclonal diversity during expansion. Nature..

[15] Zhang H, Karnoub ER, Umeda S, Chaligné R, Masilionis I, McIntyre CA (2023). Application of high-throughput single-nucleus DNA sequencing in pancreatic cancer. Nat Commun..

[16] Zafar H, Navin N, Nakhleh L, Chen K (2018). Computational approaches for inferring tumor evolution from single-cell genomic data. Curr Opin Syst Biol..

[17] Jahn K, Kuipers J, Beerenwinkel N (2016). Tree inference for single-cell data. Genome Biol..

[18] Ross EM, Markowetz F (2016). OncoNEM: inferring tumor evolution from single-cell sequencing data. Genome Biol..

[19] Malikic S, Jahn K, Kuipers J, Sahinalp SC, Beerenwinkel N (2019). Integrative inference of subclonal tumour evolution from single-cell and bulk sequencing data. Nat Commun..

[20] Malikic S, Mehrabadi FR, Ciccolella S, Rahman MK, Ricketts C, Haghshenas E (2019). PhISCS: a combinatorial approach for subperfect tumor phylogeny reconstruction via integrative use of single-cell and bulk sequencing data. Genome Res..

[21] Kimura M (1969). The number of heterozygous nucleotide sites maintained in a finite population due to steady flux of mutations. Genetics..

[22] Demeulemeester J, Dentro SC, Gerstung M, Van Loo P (2022). Biallelic mutations in cancer genomes reveal local mutational determinants. Nat Genet..

[23] Kuipers J, Jahn K, Raphael BJ, Beerenwinkel N (2017). Single-cell sequencing data reveal widespread recurrence and loss of mutational hits in the life histories of tumors. Genome Res..

[24] McPherson A, Roth A, Laks E, Masud T, Bashashati A, Zhang AW (2016). Divergent modes of clonal spread and intraperitoneal mixing in high-grade serous ovarian cancer. Nat Genet..

[25] El-Kebir M (2018). SPhyR: tumor phylogeny estimation from single-cell sequencing data under loss and error. Bioinformatics..

[26] Ciccolella S, Soto Gomez M, Patterson MD, Della Vedova G, Hajirasouliha I, Bonizzoni P (2020). gpps: an ILP-based approach for inferring cancer progression with mutation losses from single cell data. BMC Bioinformatics..

[27] Ciccolella S, Ricketts C, Soto Gomez M, Patterson M, Silverbush D, Bonizzoni P (2021). Inferring cancer progression from single-cell sequencing while allowing mutation losses. Bioinformatics..

[28] Farris JS (1977). Phylogenetic analysis under Dollo’s Law. Syst Biol..

[29] Zafar H, Tzen A, Navin N, Chen K, Nakhleh L (2017). SiFit: inferring tumor trees from single-cell sequencing data under finite-sites models. Genome Biol..

[30] Zafar H, Navin N, Chen K, Nakhleh L (2019). SiCloneFit: Bayesian inference of population structure, genotype, and phylogeny of tumor clones from single-cell genome sequencing data. Genome Res..

[31] Wagner WH. Problems in the classification of ferns. Recent Adv Bot. 1961;(1):841–4.

[32] Satas G, Zaccaria S, Mon G, Raphael BJ (2020). Scarlet: Single-cell tumor phylogeny inference with copy-number constrained mutation losses. Cell Syst..

[33] Chen Z, Gong F, Wan L, Ma L (2022). BiTSC 2: Bayesian inference of tumor clonal tree by joint analysis of single-cell SNV and CNA data. Brief Bioinforma..

[34] Sollier E, Kuipers J, Takahashi K, Beerenwinkel N, Jahn K. COMPASS: joint copy number and mutation phylogeny reconstruction from amplicon single-cell sequencing data. Nature Communications. 2023;14(1):4921. 10.1038/s41467-023-40378-8 PMC10427627 37582954

[35] Lähnemann D, Köster J, Szczurek E, McCarthy DJ, Hicks SC, Robinson MD (2020). Eleven grand challenges in single-cell data science. Genome Biol..

[36] Zaccaria S, Raphael BJ (2021). Characterizing allele-and haplotype-specific copy numbers in single cells with CHISEL. Nat Biotechnol..

[37] Leung ML, Davis A, Gao R, Casasent A, Wang Y, Sei E (2017). Single-cell DNA sequencing reveals a late-dissemination model in metastatic colorectal cancer. Genome Res..

[38] Lan F, Demaree B, Ahmed N, Abate AR (2017). Single-cell genome sequencing at ultra-high-throughput with microfluidic droplet barcoding. Nat Biotechnol..

[39] Pellegrino M, Sciambi A, Treusch S, Durruthy-Durruthy R, Gokhale K, Jacob J (2018). High-throughput single-cell DNA sequencing of acute myeloid leukemia tumors with droplet microfluidics. Genome Res..

[40] Gusfield D (1991). Efficient algorithms for inferring evolutionary trees. Networks..

[41] Singer J, Kuipers J, Jahn K, Beerenwinkel N (2018). Single-cell mutation identification via phylogenetic inference. Nat Commun..

[42] Weber LL, Sashittal P, El-Kebir M (2021). doubletD: detecting doublets in single-cell DNA sequencing data. Bioinformatics..

[43] Gurobi Optimization, LLC. Gurobi Optimizer Reference Manual. 2020. http://www.gurobi.com. Accessed 5 Mar 2021.

[44] Krapivsky PL, Redner S (2001). Organization of growing random networks. Phys Rev E..

[45] MacQueen J. Classification and analysis of multivariate observations. In: 5th Berkeley Symp. Math. Statist. Probability. University of California Press; 1967. p. 281–297.

[46] Rousseeuw PJ (1987). Silhouettes: a graphical aid to the interpretation and validation of cluster analysis. J Comput Appl Math..

[47] Hayashi A, Hong J, Iacobuzio-Donahue CA (2021). The pancreatic cancer genome revisited. Nat Rev Gastroenterol Hepatol..

[48] Greer JB, Whitcomb DC (2007). Role of BRCA1 and BRCA2 mutations in pancreatic cancer. Gut..

[49] Gerlinger M, Rowan AJ, Horswell S, Larkin J, Endesfelder D, Gronroos E (2012). Intratumor heterogeneity and branched evolution revealed by multiregion sequencing. N Engl j Med..

[50] Zhang J, Fujimoto J, Zhang J, Wedge DC, Song X, Zhang J (2014). Intratumor heterogeneity in localized lung adenocarcinomas delineated by multiregion sequencing. Science..

[51] Hiley C, de Bruin EC, McGranahan N, Swanton C (2014). Deciphering intratumor heterogeneity and temporal acquisition of driver events to refine precision medicine. Genome Biol..

[52] Stanta G, Bonin S (2018). Overview on clinical relevance of intra-tumor heterogeneity. Front Med..

[53] Sashittal P, Zaccaria S, El-Kebir M (2022). Parsimonious Clone Tree Integration in cancer. Algorithms Mol Biol..

[54] Stoler N, Nekrutenko A (2021). Sequencing error profiles of Illumina sequencing instruments. NAR Genomics Bioinforma..

[55] Köchl S, Niederstätter H, Parson W, Carracedo A (2005). DNA Extraction and Quantitation of Forensic Samples Using the Phenol-Chloroform Method and Real-Time PCR. Forensic DNA Typing Protocols.

[56] Coyne SR, Craw PD, Norwood DA, Ulrich MP (2004). Comparative analysis of the Schleicher and Schuell IsoCode Stix DNA isolation device and the Qiagen QIAamp DNA mini kit. J Clin Microbiol..

[57] Li H, Durbin R (2009). Fast and accurate short read alignment with Burrows-Wheeler transform. Bioinformatics..

[58] "Picard toolkit." Broad Institute. Broad Institute, GitHub repository. 2019. Available from: https://broadinstitute.github.io/picard.

[59] McKenna A, Hanna M, Banks E, Sivachenko A, Cibulskis K, Kernytsky A (2010). The Genome Analysis Toolkit: a MapReduce framework for analyzing next-generation DNA sequencing data. Genome Res..

[60] Cibulskis K, Lawrence MS, Carter SL, Sivachenko A, Jaffe D, Sougnez C (2013). Sensitive detection of somatic point mutations in impure and heterogeneous cancer samples. Nat Biotechnol..

[61] Poplin R, Ruano-Rubio V, DePristo MA, Fennell TJ, Carneiro MO, Van der Auwera GA, et al. Scaling accurate genetic variant discovery to tens of thousands of samples. BioRxiv. 2018;201178.

[62] Cerami E, Gao J, Dogrusoz U, Gross BE, Sumer SO, Aksoy BA (2012). The cBio Cancer Genomics Portal: An Open Platform for Exploring Multidimensional Cancer Genomics Data. Cancer Discov..

[63] Gao J, Aksoy BA, Dogrusoz U, Dresdner G, Gross B, Sumer SO, et al. Integrative analysis of complex cancer genomics and clinical profiles using the cBioPortal. Sci Signal. 2013;6(269):pl1–pl1. 10.1126/scisignal.2004088. 10.1126/scisignal.2004088 PMC4160307 23550210

[64] Pagel KA, Kim R, Moad K, Busby B, Zheng L, Tokheim C (2020). Integrated Informatics Analysis of Cancer-Related Variants. JCO Clin Cancer Informa..

[65] Hayashi A, Hong J, Iacobuzio-Donahue CA (2021). The pancreatic cancer genome revisited. Nat Rev Gastroenterol Hepatol..

[66] Demaree B, Delley CL, Vasudevan HN, Peretz CAC, Ruff D, Smith CC (2021). Joint profiling of DNA and proteins in single cells to dissect genotype-phenotype associations in leukemia. Nat Commun..

[67] LJPvd M, Hinton G (2008). Visualizing high-dimensional data using t-SNE. J Mach Learn Res..

[68] Sashittal P, Zhang H, Iacobuzio-Donahue C, Raphael B. ConDoR: Tumor phylogeny inference with a copy-number constrained mutation loss model. Zenodo. 2023. 10.5281/zenodo.8350264. 10.1186/s13059-023-03106-5 PMC10688497 38037115

[69] Leung ML, Davis A, Gao R, Casasent A, Wang Y, Sei E, et al. Data from Single-cell DNA sequencing reveals a late-dissemination model in metastatic colorectal cancer. NCBI SRA. 2017. https://www.ncbi.nlm.nih.gov/sra/?term=SRP074289. Processed data from this study was accessed from https://github.com/raphael-group/scarlet. Accessed 5 Mar 2023. 10.1101/gr.209973.116 PMC5538546 28546418

