# Supplementary material for: ConDoR: tumor phylogeny inference with a copy-number constrained mutation loss model
Source: Genome Biol. 2023 Nov 30;24:272. doi: 10.1186/s13059-023-03106-5 (PMC10688497; doi:10.1186/s13059-023-03106-5)
Supplement: Supplementary file 1 — Additional file 1. Figures, tables and text describing additional information such as proofs of theorems or additional results. [file 13059_2023_3106_MOESM1_ESM.pdf]

Additional file 1 for “ConDoR: Tumor phylogeny inference with a copy-number constrained mutation loss model”

Palash Sashittal[1], Haochen Zhang[2], Christine A. Iacobuzio-Donahue[3,4,5], and Benjamin J. Raphael[1,*]

[1]Department of Computer Science, Princeton University, NJ, USA

[2]Gerstner Sloan Kettering Graduate School of Biomedical Sciences, MSKCC, NY, USA

[3]Human Oncology and Pathogenesis Program, MSKCC, NY, USA

[4]David M. Rubenstein Center for Pancreatic Cancer Research, MSKCC, NY, USA

[5]Department of Pathology and Laboratory Medicine, MSKCC, NY, USA

[*]Correspondence: braphael@princeton.edu

Contents

A Proofs **2**

A.1 Proof to Theorem 2 in the Main text 2

A.2 Proof to Theorem 3 in the Main text 5

B MILP formulation **6**

B.1 k-Dollo completion constraints 6

B.2 Complete MILP 9

C Method parameters **11**

D Additional simulations **13**

D.1 Different read count model 13

D.2 Noise in copy number clustering 15

E Supplementary results **16**

A Proofs

A.1 Proof to Theorem 2 in the Main text

Before we start with the proof, we state a few definitions and results that would be useful in the proof.

Definition 1 (k-Dollo state tree, El-Kebir 2018 [1])**.** The k-Dollo state tree $S[k]$ is a state tree with nodes $\{0, \ldots, k+1\}$ and edges $\{0, 1\} \cup \{(1, s) | s \in \{2, \ldots, k+1\}\}$.

Definition 2 (Binary factorization)**.** Let B be a $n \times m$ multi-state character matrix for m characters with $r+1$ states $\{0, 1, \ldots, r\}$ and state tree S with 0 as the root state. The binary factorization $B' = [b'_{i,(j,s)}]$ of B is a $n \times mr$ binary matrix in which $b'_{i,(j,s)} = 1$ for $j \in [m], s \in [r]$ if $b_{i,j} = s'$ where $s \preceq_S s'$, i.e. s' is in the subtree of S rooted at s.

The following theorem from [1] relates the k-Dollo state tree with k-Dollo completion matrices.

Theorem 1 (El-Kebir 2018 [1])**.** Let $B \in \{0, \ldots, k+1\}^{n \times m}$ be a k-Dollo completion matrix. Then there exists a multi-state perfect phylogeny T_B for B such that each mutation $j \in [m]$ has the k-Dollo state tree $S[k]$.

The following theorem describes a characterization of matrices that admits a binary perfect phylogeny.

Theorem 2 ([2])**.** Binary matrix A admits a perfect phylogeny if and only if no pair of columns (j, j') that contain the three gametes $(0, 1), (1, 0)$ and $(1, 1)$.

Now, we provide the proof for Theorem 2 in the main text. We restate the theorem here for completeness and provide the proof in the following.

Theorem 3. There exists a constrained k-Dollo phylogeny T with at most k SNV losses for $A \in \{0, 1\}^{n \times m}$ and copy-number clustering $\sigma : [n] \to [p]$ if and only if there exists a k-Dollo completion $B \in \{0, \ldots, k+1\}^{n \times m}$ of A that is consistent with σ.

Proof. ($\Rightarrow$) Let T be a constrained k-Dollo phylogeny T for binary matrix A and copy-number clustering σ with at most k losses. We construct a k-Dollo completion matrix B of A and show that it is consistent with σ.

We construct B as follows. For SNV $j \in [m]$, let edges of T that induce a loss in SNV j be given by the set $\{e_{j,2}, \ldots, e_{j,q}\}$, where $q \leq k+1$ and the edges are indexed in a breadth first manner. For each leaf $v \in L(T)$ corresponding to row i in matrix A, we set $b_{i,j} = s$ if the edge $e_{j,s}$ is in the unique path from the

root $r(T)$ to v. For each $(i, j) \in [n] \times [m]$, if $a_{i,j} = 1$, we set $b_{i,j} = 1$. For the rest of the entries we set $b_{i,j} = 0$. It is easy to see that B is a k-completion of A.

Let us assume that B is not consistent with copy-number clustering σ. Then for some $j \in [m]$, either condition (1) or condition (2) of Definition 4 in the main text is violated.

Case 1: Lets say condition (1) is violated. Then, there must exist two clusters ℓ and ℓ' such that $b_{i,j} = 0$ and $b_{i',j} = 1$ for distinct cells i, i' of these clusters. Since the set of nodes labeled by the clusters ℓ and ℓ' are disjoint, the mutation j must be gained at least twice in T. However, this contradicts the premise that T is a constrained Dollo phylogeny.

Case 2: Lets say condition (2) is violated. Then, there must exist two rows i and i' such that $b_{i,j} = s$ for $s \in \{2, \ldots, k+1\}$, $b_{i',j} \neq s$ and $\sigma(i) = \sigma(i') = \ell$. Let v and v' be the leaves corresponding to the rows i and i' of B. Let $r(\ell)$ be the root of the subtree induced by the nodes labeled by ℓ in T. Consider the path from the root $r(T)$ to v_i. The node $r(\ell)$ be lie of this path. As such, we can denote this path by $P = \{r(T), \ldots, r(\ell), \ldots, v_i\}$. Since $b_{i,j} = s$ and $s \in \{2, \ldots, k+1\}$, by construction, there must exist an edge $e \in P$ that induces a loss in SNV j. Moreover, since losses can only occur between nodes of T with distinct cluster labeling, edge e most lie in the path $\{r(T), \ldots, r(\ell)\}$. Let $b_{i',j} = t \neq s$. We have three cases, (i) $t = 0$, (ii) $t = 1$ and (iii) $t \in \{2, \ldots, k+1\} \setminus \{s\}$.

Case (i): Since $\sigma(v) = \sigma(v') = \ell$ and T is a constrained Dollo phylogeny, $r(\ell)$ must lie in the unique path from $r(T)$ to v'. As such, the edge e which induces a loss of SNV j must lie on the path from $r(T)$ to v'. However since $b_{i',j} = 0$, by construction, the SNV j is neither lost or gained along the path from $r(T)$ to v', which is a contradiction.

Case (ii): Since $b_{i',j} = 1$, by construction, we have $a_{i',j} = 1$. Considering the path from $r(T)$ to v', we have an edge e at which SNV j is lost in the subpath from $r(T)$ to $r(\ell)$. The SNV must be gained in an edge preceding e in the path P. Since $a_{i',j} = 1$, the SNV must also be gained in an edge succeeding e in the path P. However, this contradicts the premise that T is a constrained Dollo phylogeny in which each SNV can be gained at most once.

Case (iii): Since $b_{i',j} = t$ and $t \in \{2, \ldots, k+1\}$, by construction, there must exist an edge e' in the path $P' = \{r(T), \ldots, r(\ell), \ldots, v'\}$ that induces a loss in SNV j. Moreover, since losses can only occur between nodes of T with distinct cluster labeling, edge e' must lie in the path $\{r(T), \ldots, r(\ell)\}$. By construction e and e' are distinct since $s \neq t$. Without loss of generality, let $s < t$. As such, the SNV must be gained once along the path $r(T)$ to the source node of e and once along the path from the target node of e to the source node of e'. However, this contradicts the premise that T is a constrained Dollo phylogeny in which each SNV can be gained at most once.

($\Leftarrow$) Let B be a k-Dollo completion matrix of A that is consistent with copy-number clustering σ. For cluster $\ell \in [p]$, let $\sigma^{-1}(\ell)$ be the set of cells in that cluster, i.e.

$$\sigma^{-1}(\ell) = \{i : \sigma(i) = \ell, i \in [n]\}.$$

We show that there exists a constrained k-Dollo phylogeny T for A and copy-number clustering σ. Equivalently, we show that there exists a state tree on the copy-number clusters such that the k-Dollo completion matrix B along with the character for the copy-number cluster admits a multi-state perfect phylogeny. Recall that k-Dollo completion matrix B admits a k-Dollo phylogeny and as such, the binary factorization B' of B with respect to the k-Dollo state tree admits a two-state perfect phylogeny.

First, we define a matrix $R \in \{0, 1, \ldots, k+1\}^{p \times m}$ which represents the mutational state of the roots of the subtrees formed by each copy number cluster. We build R from the k-Dollo completion matrix B as follows,

$$r_{\ell,j} = \begin{cases} s, & \text{if } b_{i,j} = s, \text{for all } i \in \sigma^{-1}(\ell) \text{ and } s \in \{0, 1, 2, \ldots, k+1\}, \\ 0, & \text{otherwise.} \end{cases} \tag{12}$$

We show that the $(n+p) \times m$ augmented matrix $\overline{B} = [B; R]$ admits a multi-state perfect phylogeny with k-Dollo state tree $S[k]$. This is equivalent to showing that the binary factorization $\overline{B}' = [B'; R']$ of $\overline{B}$ admits a two-state perfect phylogeny. We show this by contradiction. If $\overline{B}'$ does not admit a perfect phylogeny, let (j, s) and (j', s') be two columns of $\overline{B}'$ that have the three gametes $(0, 1), (1, 0)$ and $(1, 1)$. Note that B' admits a perfect phylogeny, since B is a k-Dollo completion matrix. As such, at least one of the rows for the three gametes must correspond to $(r'_{\ell,(j,s)}, r'_{\ell,(j',s')})$ for some cluster ℓ. However, by construction of R, there must be at least one cell in cluster ℓ that has the same gamete. This would violate the 3-gamete condition for B', which is a contradiction.

Now we show that R' form roots of subtrees in the two-state perfect phylogeny for $\overline{B}'$. Note that when a perfect phylogeny exists, it is unique [3]. Moreover, B' admits a two-state perfect phylogeny since B is a k-Dollo completion matrix. As such, we only need to show that R' are roots of subtrees in the two-state perfect phylogeny for B'. Consider a cluster ℓ. By construction of R, it is clear that for any mutation j, the state $r_{\ell,j}$ is the lowest common ancestor of the states of the cells $i \in \sigma(\ell)$ with respect to the state tree $S[k]$, i.e. $r_{\ell,j} \coloneqq LCA_{S[k]}(\{b_{i,j} \mid i \in \sigma^{-1}(\ell)\})$. We only need to make sure that the subtrees formed by the 1-sets are either related by containment or disjoint of the subtrees defined by the clusters. By contradiction, suppose there is a mutation j for which the 1-set intersects with the two clusters ℓ and ℓ' such that $r_{\ell,j} = 0$

and $r_{\ell',j} = 0$. However, this would mean that there are two clusters ℓ and ℓ' where the mutation j is gained, which contradicts the premise that matrix B is consistent with the copy-number clustering σ.

□

A.2 Proof to Theorem 3 in the Main text

We restate the theorem here for completeness and provide a proof in the following.

Theorem 4. The CkDP-RC problem is NP-hard, even for $k = 0$.

Proof. We show this by reduction from the *Flip problem* [4] which is known to be NP-complete and is stated as follows:

Problem 1 (Flip problem)**.** Given a binary matrix D and an integer $c \in \mathbb{N}$, does there exist a binary matrix B such that (i) B differs from D by at most c entries and (ii) B admits a perfect phylogeny.

Given a instance (D, c) of the Flip problem we construct an instance of the CkDP-RC problem with $k = 0$ as follows. We put all the cells $i \in [n]$ in the same copy-number cluster, i.e. $\sigma(i) = 1$ for all $i \in [n]$. The variant read count matrix Q and total read count matrix R as built as follows. For each entry $(i, j) \in [n] \times [m]$ with $d_{i,j} = 0$, we set $q_{i,j} = q_0$ and $r_{i,j} = r_0$, where q_0 and r_0 are chosen such that $\Pr(q_0 \mid r_0, a_{i,j} = 0) = \mu > 0.5$. For each entry $(i, j) \in [n] \times [m]$ with $d_{i,j} = 1$, we set $q_{i,j} = q_0$ and $r_{i,j} = r_0$, where q_0 and r_0 are chosen such that $\Pr(q_0 \mid r_0, a_{i,j} = 1) = \mu > 0.5$. Clearly this construction can be completed in polynomial time.

The likelihood of A is given by

$$\Pr(Q \mid R, A) = \prod_{i=1}^{n}\prod_{j=1}^{m}(\Pr(q_1 \mid r_1, a_{i,j}))^{\mathbf{1}(d_{i,j}=1)}(\Pr(q_0 \mid r_0, a_{i,j}))^{\mathbf{1}(d_{i,j}=0)},$$

where $\mathbf{1}(\cdot)$ is the indicator function. Let c' be the number of entries where D and A differ. Then we have

$$\Pr(Q \mid R, A) = \mu^{nm-c'}(1-\mu)^{c'}. \tag{13}$$

We show that the Flip problem instance (D, c) has a solution if and only if the corresponding CkDP-RC has a solution with likelihood

$$\Pr(Q \mid R, A) \geq \mu^{nm-c}(1-\mu)^{c}.$$

($\Rightarrow$) Let B be the solution to the Flip problem instance (D, c). From Eq. 13, the likelihood for mutation matrix $A = B$ is given by $\mu^{nm-c}(1-\mu)^{c}$. Moreover, B is a perfect phylogeny and therefore it is also a constrained 0-Dollo phylogeny with the constructed copy-number clustering σ.

($\Leftarrow$) Let A be the solution to the CkDP-RC problem such that

$$\Pr(Q \mid R, A) \geq \mu^{nm-c}(1-\mu)^c. \tag{14}$$

Then, suppose A differs from D in $c' > c$ entries. Then from Eq. 13 we have

$$\Pr(Q \mid R, A) = \mu^{nm-c'}(1-\mu)^{c'} < \mu^{nm-c}(1-\mu)^c,$$

since $\mu > 0.5$. But this contradicts Eq. 14. Hence A must differ from D in at most c entries and thus is a solution to the Flip problem instance (D, c). □

B MILP formulation

B.1 k-Dollo completion constraints

Definition 3 in the main text provides $O(k^4)$ distinct 3×2 submatrices that must be avoided in a k-Dollo completion matrix B. Incorporating this in the MILP will require $O(n^3m^2k^4)$ constraints which is infeasible for large values of n, m and k. We instead employ an alternate characterization of k-Dollo completion matrices derived from the work of [5]. In this characterization, for a given matrix $B \in \{0, \ldots, k+1\}^{n \times m}$, we define the $n \times m(k+1)$ *extended binary matrix* B' such that B is a k-Dollo completion matrix for some mutation matrix A if and only if B' admits a perfect phylogeny.

Definition 3. The extended binary matrix $B' \in 0, 1^{n \times m(k+1)}$ is obtained from the k-completion matrix B as follows: for each entry $b_{i,j}$ we have the entry b'_{i,j^+} and k entries b'_{i,j^-_s} (for $s \in \{2, \ldots, k+1\}$) such that (i) if $b_{i,j} = 0$ then $b'_{i,j^+} = 0$ and $b'_{i,j^-_s} = 0$ for $s \in \{2, \ldots, k+1\}$, (ii) if $b_{i,j} = 1$ then $b'_{i,j^+} = 1$ and $b'_{i,j^-_s} = 0$ for $s \in \{2, \ldots, k+1\}$, (iii) if $b_{i,j} = s$ for $s > 1$ then $b'_{i,j^+} = 1$, $b'_{i,j^-_s} = 1$ and $b'_{i,j^-_t} = 0$ for $t \in \{2, \ldots, k+1\} \setminus \{s\}$.

Theorem 3.2 in [6] states the following.

Lemma 1. Matrix $B \in \{0, \ldots, k+1\}$ is a k-Dollo completion matrix of some mutation matrix A if and only if the corresponding extended binary matrix B' admits a perfect phylogeny.

We now encode the extended binary matrix B' obtained from the k-completion matrix B of the mutation matrix A in our MILP formulation. Note that $b'_{i,j^+} = 1$ if and only if $b_{i,j} \geq 1$. As such, the entries b'_{i,j^+} are modeled by the variable $x_{i,j}$. Further, $b'_{i,j^-_s} = 1$ if and only if $b_{i,j} = s$ for $s \in \{2, \ldots, k+1\}$. As such, the entries b'_{i,j^-_s} are modeled by $c_{\sigma(i),j,s}$. We use the *set inclusion and disjointness* (SID) formulation described in [7] to enforce that the extended binary matrix B' is a perfect phylogeny. The SID formulation is based on the following theorem from [2].

Theorem 5 ([2]). Let $A = [a_{i,j}]$ be a binary $n \times m$ matrix and let $I(j) \coloneqq \{i : a_{i,j} = 1\}$ be the set of 1-indices in column j. Then A is a perfect phylogeny if and only if for each pair of columns j and j', we have $I(j) \subseteq I(j')$ or $I(j') \subseteq I(j)$ or $I(j) \cap I(j') = \emptyset$.

The above theorem states that a binary matrix admits a perfect phylogeny if and only if for any two columns j and j', their 1-sets (i.e. $I(j)$ and $I(j')$) must be either be related by containment or be disjoint.

Note the extended binary matrix B' has the following $m(k+1)$ columns.

$$\{j^+ : j \in [m]\} \cup \{j_s : j \in [m], s \in \{2, \dots, k+1\}\}$$

. Let the set of loss states $\{2, \dots, k+1\}$ be denoted by $\mathcal{L}_k$. We enforce the conditions described in Theorem 5 on the extended binary matrix B' as follows.

k-Dollo completion constraints First, we introduce continuous variables that indicate containment between 1-sets of different columns in the extended binary matrix B'. We introduce the following set of variables.

1. $y^{(0)}_{j,j',s,s'}$ for $j, j' \in [m]$ and $s, s' \in \mathcal{L}_{\|}$, which will be 1 only if 1-set of column j_s contains 1-set of column $j'_{s'}$.
2. $y^{(1)}_{j,j',s'}$ for $j, j' \in [m]$ and $s \in \mathcal{L}_{\|}$, which will be 1 only if 1-set of column j^+ contains 1-set of column $j'_{s'}$.
3. $y^{(2)}_{j,j',s}$ for $j, j' \in [m]$ and $s \in \mathcal{L}_{\|}$, which will be 1 only if 1-set of column j_s contains 1-set of column j'^+.
4. $y^{(3)}_{j,j'}$ for $j, j' \in [m]$, which will be 1 only if 1-set of column j^+ contains 1-set of column j'^+.

We enforce this using the following constraints.

$$y^{(0)}_{j,j',s,s'} \leq 1 - c_{\ell,j',s'} + c_{\ell,j,s}, \qquad \text{for all } \ell \in [p], j, j' \in [m], j \neq j', s, s' \in \mathcal{S}_k, \tag{15}$$

$$y^{(1)}_{j,j',s'} \leq 1 - c_{\ell,j',s'} + \hat{x}_{\ell,j}, \qquad \text{for all } \ell \in [p], j, j' \in [m], j \neq j', s' \in \mathcal{S}_k, \tag{16}$$

$$y^{(2)}_{j,j',s} \leq 1 - (g^{(1)}_{\ell,j'} + \sum_{s'=1}^{k} c_{\ell,j',s'}) + c_{\ell,j,s}, \quad \text{for all } \ell \in [p], j, j' \in [m], j \neq j', s \in \mathcal{S}_k, \tag{17}$$

$$y^{(3)}_{j,j'} \leq 1 - x_{i,j'} + x_{i,j}, \qquad \text{for all } i \in [n], j, j' \in [m], j \neq j'. \tag{18}$$

Next, we introduce variables to indicate if the 1-set of two columns are disjoint.

1. $z^{(0)}_{j,j',s,s'}$ for $j, j' \in [m]$ and $s, s' \in \mathcal{L}_{\|}$, which will be 1 only if 1-sets of column j_s and column $j'_{s'}$ are disjoint.

2. $z^{(1)}_{j,j',s'}$ for $j, j' \in [m]$ and $s \in \mathcal{L}_{\|}$, which will be 1 only if 1-sets of column j^+ and column $j'_{s'}$ are disjoint.

3. $z^{(2)}_{j,j'}$ for $j, j' \in [m]$, which will be 1 only if 1-sets of column j^+ and column j'^+ are disjoint.

We enforce this using the following constraints.

$$z^{(0)}_{j,j',s,s'} \leq 2 - c_{\ell,j,s} - c_{\ell,j',s'}, \qquad \text{for all } \ell \in [p], j, j' \in [m], j < j', s, s' \in \mathcal{S}_k, \tag{19}$$

$$z^{(1)}_{j,j',s'} \leq 2 - c_{\ell,j',s'} - (g^{(1)}_{\ell,j} + \sum_{s=1}^{k} c_{\ell,j,s}), \quad \text{for all } \ell \in [p], j, j' \in [m], j \neq j', s' \in \mathcal{S}_k, \tag{20}$$

$$z^{(2)}_{j,j'} \leq 2 - x_{i,j'} - x_{i,j}, \qquad \text{for all } i \in [n], j, j' \in [m], j < j'. \tag{21}$$

Following Theorem 5, we require the following conditions to attain a perfect phylogeny.

$$y^{(0)}_{j,j',s,s'} + y^{(0)}_{j',j,s',s} + z^{(0)}_{j,j',s,s'} \geq 1, \quad \text{for all } j, j' \in [m], j < j', s, s' \in \mathcal{S}_k, \tag{22}$$

$$y^{(1)}_{j,j',s'} + y^{(2)}_{j',j,s'} + z^{(1)}_{j,j',s'} \geq 1, \quad \text{for all } j, j' \in [m], j \neq j', s, s' \in \mathcal{S}_k, \tag{23}$$

$$y^{(3)}_{j,j'} + y^{(3)}_{j',j} + z^{(2)}_{j,j'} \geq 1, \quad \text{for all } j, j' \in [m], j < j'. \tag{24}$$

This formulation contains $O(nm^2k^2)$ constraints with $O(m^2k^2)$ continuous variables.

B.2 Complete MILP

For completeness sake, we describe the complete MILP here.

$$
\begin{aligned}
\max\ & \sum_{i=1}^{n}\sum_{j=1}^{m}\Big(a_{i,j}\log\Pr(q_{i,j}\mid r_{i,j},a_{i,j}=1)+ \\
& \qquad\qquad (1-a_{i,j})\log\Pr(q_{i,j}\mid r_{i,j},a_{i,j}=0)\Big) \\
\text{s.t. } & a_{i,j}+\sum_{s=2}^{k+1}c_{\sigma(i),j,s}\le 1, && \forall i\in[n],j\in[m], \\
& x_{i,j}=a_{i,j}+\sum_{s=2}^{k+1}c_{\sigma(i),j,s} && \forall i\in[n],j\in[m], \\
& g^{(0)}_{\ell,j}\ge 1-x_{i,j}, && \forall i\in\sigma^{-1}(\ell),\ell\in[p],j\in[m], \\
& g^{(0)}_{\ell,j}\le|\sigma^{-1}(\ell)|-\sum_{i\in\sigma^{-1}(\ell)}x_{i,j}, && \forall \ell\in[p],j\in[m], \\
& g^{(1)}_{\ell,j}\ge a_{i,j}, && \forall i\in\sigma^{-1}(\ell),\ell\in[p],j\in[m], \\
& g^{(1)}_{\ell,j}\le\sum_{i\in\sigma^{-1}(\ell)}a_{i,j}, && \forall \ell\in[p],j\in[m], \\
& g_{\ell,j}\le g^{(0)}_{\ell,j},\ g_{\ell,j}\le g^{(1)}_{\ell,j},\ g_{\ell,j}\ge g^{(0)}_{\ell,j}+g^{(1)}_{\ell,j}-1, && \forall \ell\in[p],j\in[m], \\
& \sum_{\ell=1}^{p}g_{\ell,j}\le 1, && \forall j\in[m], \\
& y^{(0)}_{j,j',s,s'}\le 1-c_{\ell,j',s'}+c_{\ell,j,s}, && \forall \ell\in[p],j,j'\in[m],j\neq j',s,s'\in\mathcal{S}_k, \\
& y^{(1)}_{j,j',s'}\le 1-c_{\ell,j',s'}+\hat{x}_{\ell,j}, && \forall \ell\in[p],j,j'\in[m],j\neq j',s'\in\mathcal{S}_k, \\
& y^{(2)}_{j,j',s}\le 1-(g^{(1)}_{\ell,j'}+\sum_{s'=2}^{k+1}c_{\ell,j',s'})+c_{\ell,j,s}, && \forall \ell\in[p],j,j'\in[m],j\neq j',s\in\mathcal{S}_k, \\
& y^{(3)}_{j,j'}\le 1-x_{i,j'}+x_{i,j}, && \forall i\in[n],j,j'\in[m],j\neq j', \\
& z^{(0)}_{j,j',s,s'}\le 2-c_{\ell,j,s}-c_{\ell,j',s'}, && \forall \ell\in[p],j,j'\in[m],j<j',s,s'\in\mathcal{S}_k, \\
& z^{(1)}_{j,j',s'}\le 2-c_{\ell,j',s'}-(g^{(1)}_{\ell,j}+\sum_{s=2}^{k+1}c_{\ell,j,s}), && \forall \ell\in[p],j,j'\in[m],j\neq j',s'\in\mathcal{S}_k, \\
& z^{(2)}_{j,j',s,s'}\le 2-x_{i,j'}-x_{i,j}, && \forall i\in[n],j,j'\in[m],j<j', \\
& y^{(0)}_{j,j',s,s'}+y^{(0)}_{j',j,s,s'}+z^{(0)}_{j,j',s,s'}\ge 1, && \forall j,j'\in[m],j<j',s,s'\in\mathcal{S}_k, \\
& y^{(1)}_{j,j',s'}+y^{(2)}_{j',j,s'}+z^{(1)}_{j,j',s'}\ge 1, && \forall j,j'\in[m],j\neq j',s,s'\in\mathcal{S}_k, \\
& y^{(3)}_{j,j'}+y^{(3)}_{j',j}+z^{(2)}_{j,j'}\ge 1, && \forall j,j'\in[m],j<j',
\end{aligned}
$$

$$
\begin{aligned}
&a_{i,j} \in \{0,1\}, && \forall i \in [n], j \in [m],\\
&c_{\ell,j,s} \in \{0,1\}, && \forall \ell \in [p], j \in [m], s \in \mathcal{S}_k,\\
&g_{\ell,j}, g^{(0)}_{\ell,j}, g^{(1)}_{\ell,j}, \hat{x}_{\ell,j} \in [0,1], && \forall \ell \in [p], j \in [m],\\
&y^{(0)}_{j,j',s,s'}, y^{(1)}_{j,j',s,s'}, y^{(2)}_{j,j',s,s'}, y^{(3)}_{j,j',s,s'} \in [0,1], && \forall j, j' \in [m], j \neq j', s' \in \mathcal{S}_k,\\
&z^{(0)}_{j,j',s,s'}, z^{(1)}_{j,j',s,s'}, z^{(2)}_{j,j',s,s'} \in [0,1], && \forall j, j' \in [m], j < j', s' \in \mathcal{S}_k.
\end{aligned}
$$

C Method parameters

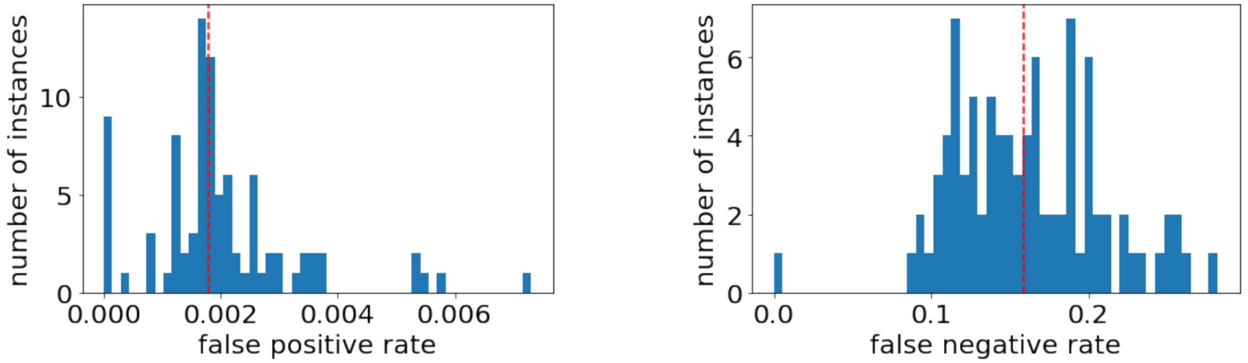

(a) (b)

Figure S1: Histogram of the (a) false positive rate α and the (b) false negative rate β in the observed mutation matrix A' of simulated instances. Red lines show the median false positive rate (0.0018) and median false negative rate (0.158).

Here we provide the precise commands used to run competing methods for benchmarking on the simulated instances. Methods such as SPhyR, SCITE and SiFit take error rates, i.e. false positive rate and false negative rate, as input during inference. For simulated data, we empirically estimate the false positive rate and false negative rates (Figure S1) in the observed mutation matrix A' obtained in the simulations. We provide the aforementioned methods with the median false positive rate $\alpha = 0.0018$ and median false negative rate $\beta = 0.158$ as input. Description of keywords used in the command-line arguments are provided in Table S1. Note that ConDoR is provided the accurate copy number clusters in the simulations as input, and SCARLET is provided the accurate copy number clusters and the ground-truth copy number tree in the simulations as input.

ConDoR We run ConDoR with both false positive and false negative sequencing error rates set to 0.001.

```
python condor.py -i ${input_clustering}
-a ${params_fp} -b ${params_fn} -k ${k}
-r ${input_total_readcounts} -v ${input_variant_readcounts}
-o ${output_prefix}
```

SCARLET We run SCARLET with the copy-number tree as well as the copy-number profiles from the simulations as input.

	command-line keyword	description
n	`${num_cells}$`	number of cells
m	`${num_mutations}$`	number of mutations
A'	`${input_character_matrix}`	observed mutation matrix
Q	`${input_variant_readcounts}`	variant allele read count matrix
R	`${input_total_readcounts}`	total read count matrix
σ	`${input_clustering}$`	copy-number clustering
(Q, R)	`${input_combined_readcount_file}$`	single file containing both Q and R
k	`${k}`	maximum losses allowed for any mutation

Table S1: Description of the keywords used in the command-line arguments.

```
python2 scarlet.py ${input_combined_readcount_file} ${copy_number_tree_file}
${output_prefix}
```

SPhyR We run SPhyR with the flag -lT (which sets the number of distinct rows in the inferred mutation matrix) set to $m + p$ since that is the maximum number of events that can occur in the phylogeny.

```
./kDPFC ${input_character_matrix} ${output_filename}
-a 0.0018 -b 0.158 -k ${k}
-lT ${params_lT} -lC ${params_lC}
```

SCITE We run SCITE for 1000000 iterations. Since we do not have any homozygous mutations in the simulations, we set -cc flag to 0.

```
scite -i ${input_character_matrix} -n ${num_mutations}$ -m ${num_cells}$
-o ${output_prefix}$ -a -cc 0 -l 1000000 -r 5
-fd 0.0018 -fn 0.158 0 -max_treelist_size 1
```

SiFit We run SiFit for 1000000 iterations.

```
java -jar sifit -ipMat ${input_character_matrix}
-m ${num_cells} -n ${num_mutations}$
-df 0 -l 1000000 -r 1 -fp 0.0018 -fn 0.0158
```

D Additional simulations

We evaluate the performance and robustness of ConDoR towards (i) misspecification of the read count model and (ii) noise in the copy number clustering on simulations from a previous publication [8] that are publicly available at `https://github.com/raphael-group/scarlet/tree/master/data/simulations`. We describe the procedure employed by Satas *et al.* [8] to generate these simulations for completeness. Satas *et al.* [8] simulated 50 datasets, each containing $n = 100$ cells, $m = 20$ mutations and $p = 4$ copy number clones. The set of losses supported between each pair of copy number clones were randomly drawn from a Poisson distribution with rate $0.2 \times m$. To simulate the topology of the phylogeny, $m + p + 1 = 25$ vertices were randomly assigned to the trunk or one of the p copy number clones. The vertices that were assigned to the trunk where joined in a linear path and the other vertices were joined to the trunk in such a way that vertices in the same copy number clone formed connected subtrees. Mutations were assigned to the 20 edges that did not correspond to change in copy number state and losses were assigned to an edge with probability 0.5 if the loss is supported and present in the parent vertex. Finally, the $n = 100$ cells were randomly assigned to the vertices of the simulated tree.

Satas et al. [8] simulate the read count data as follows. The total read counts are drawn from a Poisson distribution with mean of 100 to simulate $100\times$ coverage. When the mutation is present in a cell, the variant read counts are drawn from a Binomial distribution with rate of success given by $\max\{\text{Beta}(0.25, 0.25), 0.001\}$ and when the mutation is absent in a cell, the variant read counts are drawn from a Binomial distribution with rate of success of 0.001.

D.1 Different read count model

To investigate the effect of the read count model on the performance ConDoR, we use two sets of simulations, (i) where the read counts are simulated using the negative binomial model described in the 'Simulation details' Section in the main text and (ii) where the read counts are simulated using the binomial model used by Satas *et al.* [8].

Case (i) The results on the first set of simulations show that ConDoR and SCARLET are the top performing methods (Figure S2). We see that ConDoR has very similar performance compared to SCARLET (median 0.007 and 0.0065, respectively) in terms of the normalized mutation matrix error while the pairwise ancestral relation accuracy of ConDoR is higher than SCARLET (median 0.87 vs 0.84).

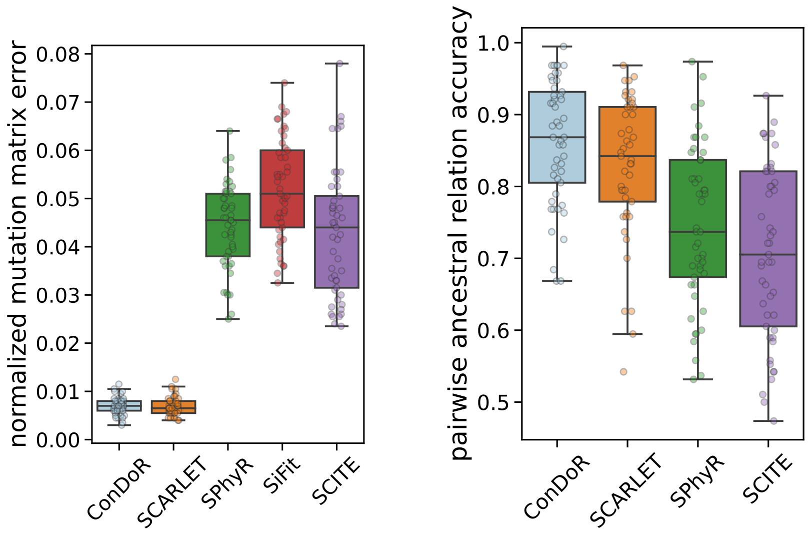

(a) (b)

Figure S2: (a) Normalized mutation matrix error and (b) pairwise ancestral relation accuracy for each method on simulations from Satas et al. [8], where the read counts were simulated using the negative binomial model (related to the Section 'Evaluation on simulated data' in the main text).

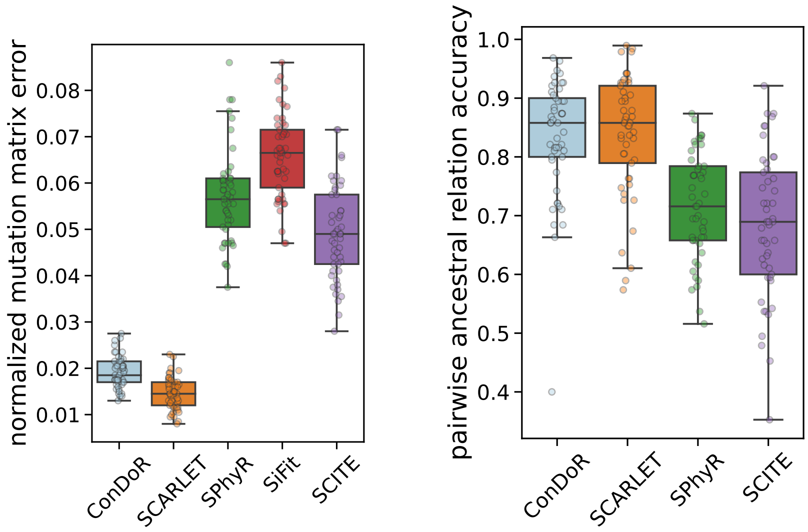

(a) (b)

Figure S3: (a) Normalized mutation matrix error and (b) pairwise ancestral relation accuracy for each method on simulations from Satas et al. [8], where the read counts were simulated using the negative binomial model (related to the Section 'Evaluation on simulated data' in the main text).

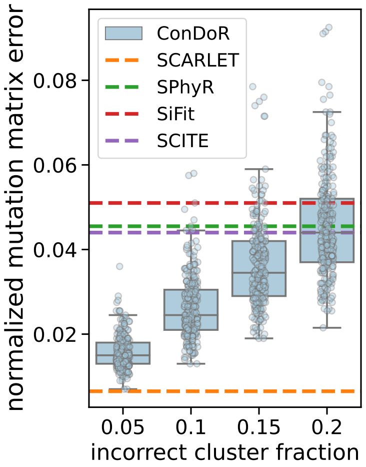

(a)

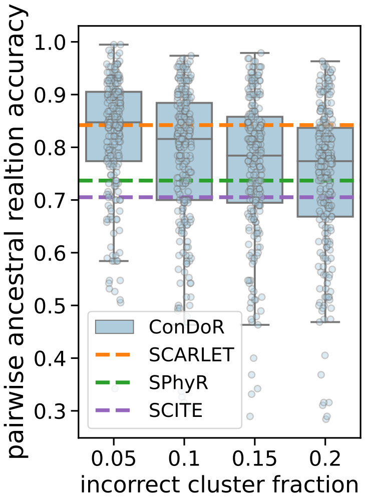

(b)

Figure S4: (a) Normalized mutation matrix error and (b) pairwise ancestral relation accuracy of ConDoR for increasing fraction h of cells with incorrect copy number clusters in the simulated instances. Dashed lines show the median performance of the other methods.

Case (ii) Figure S3 shows the results on the simulations from Satas et al. [8]. Under this setting, ConDoR outperforms all the methods except SCARLET. While the pairwise ancestral relation accuracy of SCARLET and ConDoR is very similar (median 0.8578 and 0.8578, respectively), we find that SCARLET outperforms ConDoR (median 0.0145 vs 0.0185) in terms of the mutation matrix error. However, this is expected because SCARLET is given the copy number clustering and the ground-truth copy number tree as input while ConDoR is only given the copy number clustering as input. Moreover, we use the same read count model to generate these simulations as the one used by SCARLET during inference.

D.2 Noise in copy number clustering

We evaluate the robustness of ConDoR by adding noise in the copy number clustering of the simulations described above that used the negative binomial read count model (Case (i) described in Section D.1). Let $h \in \{0.05, 0.1, 0.15, 0.2\}$ be the fraction of cells for which we provide incorrect cluster information in the simulations. We uniformly at random pick h fraction of cells to assign incorrect copy number states. For each of the picked cells we assign one of the $p-1$ incorrect copy number states that is drawn uniformly at random. We simulate 5 instances for each value of h. Figure S4 shows that ConDoR is robust to errors in

the copy number clusters. In fact, even when 20% of the cells have incorrect copy number state ($h = 0.2$), ConDoR has lower normalized mutation matrix error (meidan 0.044) and higher pairwise ancestral relation accuracy (median 0.774) compared to SPhyR (0.046 and 0.737), SiFit (0.051; pairwise ancestral relation accuracy is not applicable for SiFit) and SCITE (0.044 and 0.705).

E Supplementary results

We have the following supplementary figures and tables.

- Figure S5 shows the runtime of ConDoR, SCARLET, SPhyR, SiFit and SCITE on the simulation instances ("Evaluation on simulated data" section in the Main text).
- Figure S6 shows the distribution of length and coverage of the 596 amplicons used in the PDAC study ("Multi-region Pancreatic ductal adenocarcinoma data" section in the Main text).
- Figure S7 shows the phylogeny constructed using COMPASS and SPhyR on the PDAC dataset ("Multi-region Pancreatic ductal adenocarcinoma data" section in the Main text).
- Figure S8 shows the phylogeny constructed using SCITE and SCARLET on metastatic CRC data ("Metastatic colorectal cancer data" section in the Main text).
- Figure S9 shows the Silhouette score for increasing number of clusters in the k-means clustering of cells in the PDAC data and the TSNE plot showing the clustering with the best Silhouette score ("Multi-region Pancreatic ductal adenocarcinoma data" section in the Main text).

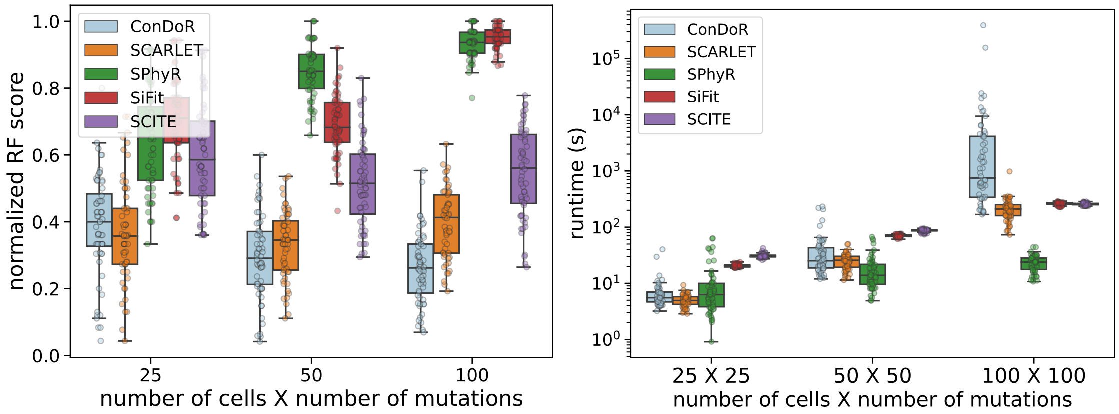

(a) **(b)**

Figure S5: (a) Normalized Robinson-Foulds distance of the phylogenies inferred by all the methods from the ground-truth phylogeny. (b)Computational time taken by all the methods on simulated instances ("Evaluation on simulated data" section in the Main text).

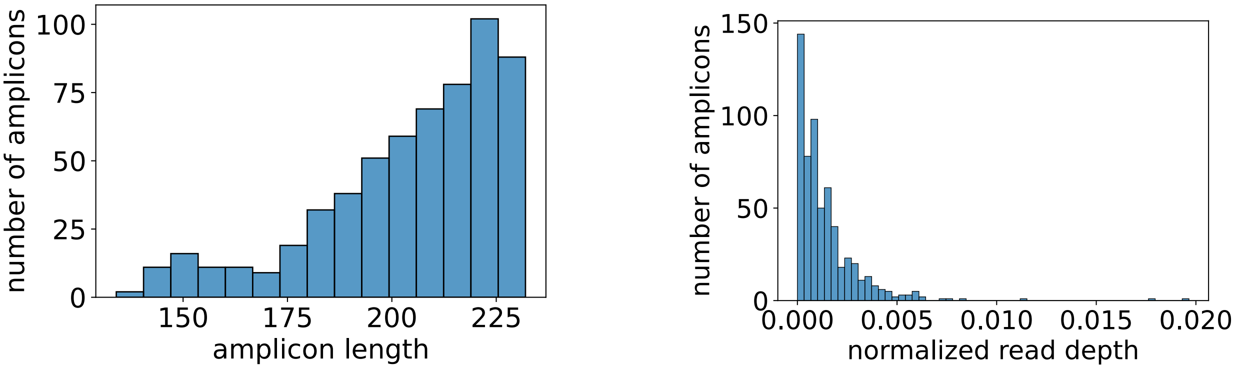

(a) **(b)**

Figure S6: Histogram of (a) length and (b) normalized read depth for the 596 amplicons used for targeted single-cell sequencing in the PDAC study ("Multi-region Pancreatic ductal adenocarcinoma data" section in the Main text). The complete panel covers 121743 bps across the genome.

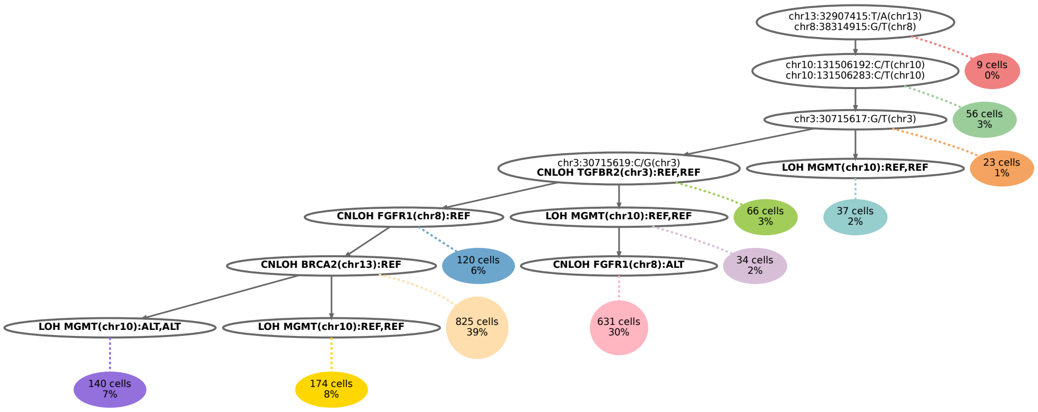

(a)

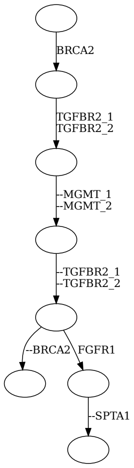

(b)

Figure S7: Phylogeny inferred by (a) COMPASS [9] and (b) SPhyR [1] on the PDAC data. COMPASS infers 8 independent LOH events across 4 genes (MGMT, BRCA2, FGFR1, TGFBR2), while SPhyR infers the loss of 6 out of the 7 mutations ("Multi-region Pancreatic ductal adenocarcinoma data" section in the Main text).

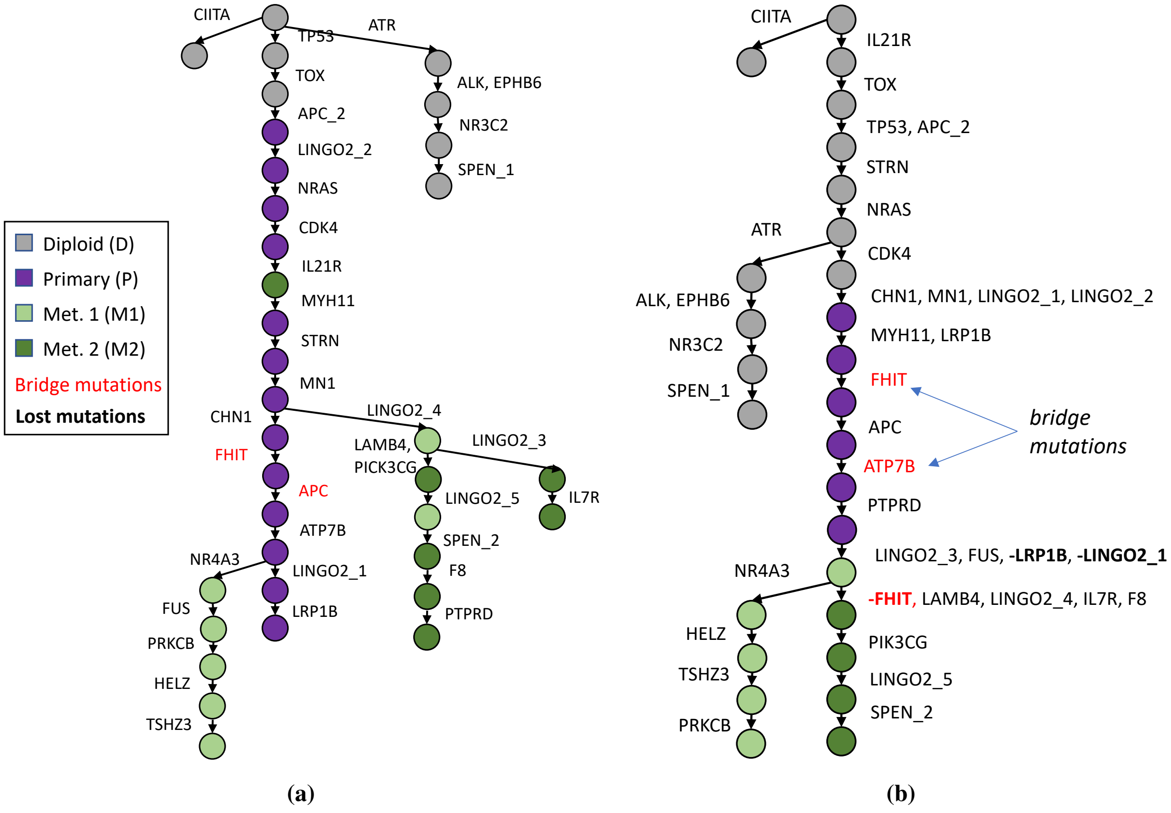

Figure S8: Phylogeny inferred by (a) SCITE [10] and (b) SCARLET [8] on the metastatic colorectal cancer data ("Metastatic colorectal cancer data" section in the Main text).

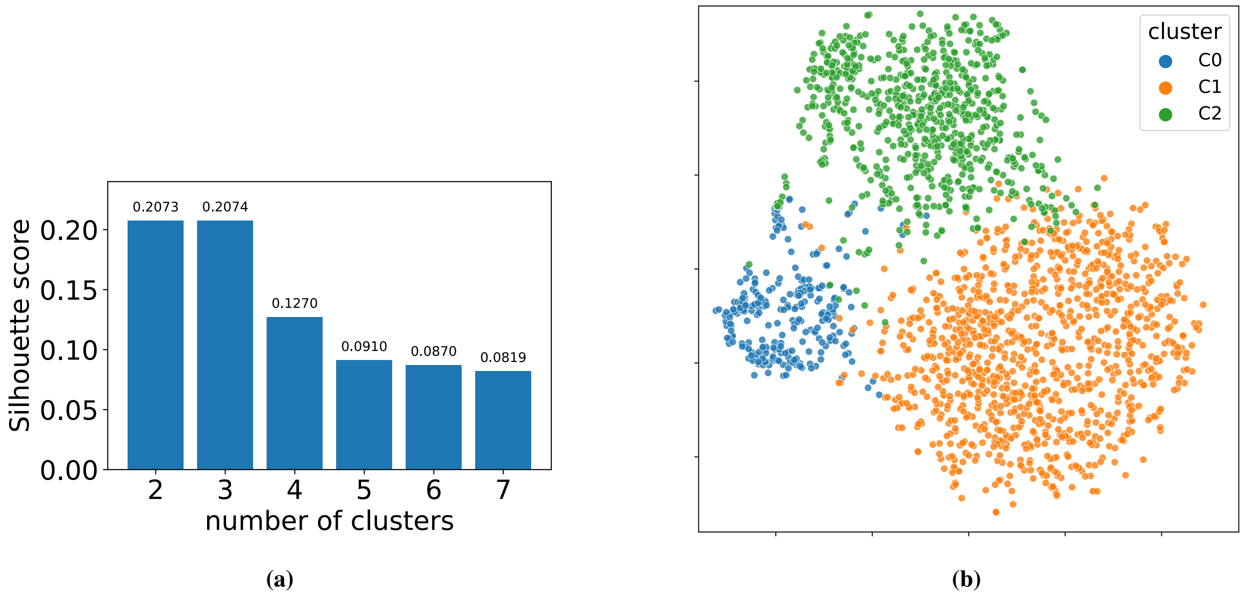

Figure S9: (a) Silhouette score for increasing number of clusters in k-means clustering of cells using the gene-level average normalized read depth $\bar{R}^G$ for the PDAC dataset. (b) tSNE plot showing the k-means clustering (that has 3 clusters) with the highest Silhouette score (0.96). Related to "Multi-region Pancreatic ductal adenocarcinoma data" section in the Main text.

References

[1] Mohammed El-Kebir. SPhyR: tumor phylogeny estimation from single-cell sequencing data under loss and error. *Bioinformatics*, 34(17):i671–i679, 2018.

[2] Dan Gusfield. Efficient algorithms for inferring evolutionary trees. *Networks*, 21(1):19–28, 1991.

[3] Dan Gusfield. Algorithms on stings, trees, and sequences: Computer science and computational biology. *Acm Sigact News*, 28(4):41–60, 1997.

[4] Duhong Chen, Oliver Eulenstein, David Fernández-Baca, and Michael Sanderson. Supertrees by flipping. In *International Computing and Combinatorics Conference*, pages 391–400. Springer, 2002.

[5] Simone Ciccolella, Mauricio Soto Gomez, Murray D Patterson, Gianluca Della Vedova, Iman Hajirasouliha, and Paola Bonizzoni. gpps: an ilp-based approach for inferring cancer progression with mutation losses from single cell data. *BMC bioinformatics*, 21(1):1–16, 2020.

[6] Paola Bonizzoni, Simone Ciccolella, Gianluca Della Vedova, and Mauricio Soto. Beyond perfect phylogeny: Multisample phylogeny reconstruction via ilp. In *Proceedings of the 8th ACM International Conference on Bioinformatics, Computational Biology, and Health Informatics*, pages 1–10, 2017.

[7] Markus Chimani, Sven Rahmann, and Sebastian Böcker. Exact ilp solutions for phylogenetic minimum flip problems. In *Proceedings of the First ACM International Conference on Bioinformatics and Computational Biology*, pages 147–153, 2010.

[8] Gryte Satas, Simone Zaccaria, Geoffrey Mon, and Benjamin J Raphael. Scarlet: Single-cell tumor phylogeny inference with copy-number constrained mutation losses. *Cell systems*, 10(4):323–332, 2020.

[9] Etienne Sollier, Jack Kuipers, Niko Beerenwinkel, Koichi Takahashi, and Katharina Jahn. Joint copy number and mutation phylogeny reconstruction from single-cell amplicon sequencing data. *bioRxiv*, 2022.

[10] Katharina Jahn, Jack Kuipers, and Niko Beerenwinkel. Tree inference for single-cell data. *Genome biology*, 17(1):1–17, 2016.
