# Supplementary material for: ConDoR: tumor phylogeny inference with a copy-number constrained mutation loss model
Source: Genome Biol. 2023 Nov 30;24:272. doi: 10.1186/s13059-023-03106-5 (PMC10688497; doi:10.1186/s13059-023-03106-5)
Supplement: Supplementary file 2 — Additional file 2. Peer review history. [file 13059_2023_3106_MOESM2_ESM.docx]

**Review history**

**First round of review**

**Reviewer 1**

Sashittal et al. propose a new method to infer tumor phylogeny while taking into account SNPs, SNVs, and CNAs. The approach is based on a new problem the define and and ILP-based solution.

The paper reads very well, is mostly very clear, and addresses an impactful problem. Most assumptions are very clear and the model appears sensible. The performance of the method on the real data seems ver promising. In general, I have high enthusiasm for this paper. Below, I present several questions and suggestions. Some of these are best addressed using new experiments but I don't believe authors can be expected to perform those experiments (which are nontrivial). However, I do strongly suggest to the authors that they consider performing these additional experiments.

## Conceptual questions

I had several conceptual questions.

1. In the likelihood function (1) I see no term that penalizes a mutation. That is, we never go over branches and asses the likelihood of a mutation. I understand the problem is constrained by other things (e.g., the Dolo constraints). But again, there is no explicit term that penalizes more/less mutations. Two scenarios, with different numbers of mutations, but exactly the same read count likelihood would be in a tie. Don't we need to choose the one with fewer mutations? If I am misunderstanding, please clarify. If this is by choice, please elaborate on why. If this is a limitation, please mention it (perhaps as future work).
2. (small point) I assumed that copy-number clustering is considering all CNAs across the genomes and summarizes them into a single clustering. Would be good to say this clearly upfront.
3. A shortcoming of the paper is that the method relies on two assumptions (which is fine) but there is no in-depth discussion of those assumptions.

Assumption 1) In particular, you need to explain why the CNV losses are allowed only among different copy-number clusters. We need a bit more biological mechanistic justification here. On the other hand, if there is no biological reason and the choice is mostly to ease computations, that's fine too, but it needs to be stated. This assumption seems fundamental and deserves more room.

Assumption 2) I would like to hear a bit more about why clusters of copy-number

should arise only once. For one region, I don't see how this is reasonable. But perhaps clustering across the genome makes this reasonable. I am pretty sure there is a reason but more discussion is needed.

## Experimental results

1. I am only mildly enthusiastic about the simulation results. The reason is that the authors have simulated under the exact set of assumptions provided and then have shown that the method works. This tells me relatively little. It's valuable but it doesn't tell me if the method is robust to violations of the assumptions. In my mind, the ultimate test is whether the model is good not whether the method is able to maximize the score defined under the model.

 In particular, it seems the method is given the correct copy number clusters. What if those are wrong?

 Would the most be robust if the counts are not drawn from the negative binomial distribution?

**Suggestion:** For (1), my suggestion is that the authors use a simulated dataset from a *previous*

publication, probably not fully compatible with their model, to also test their data.

To summarize, experiments that show robustness to model violations and incorrect input (esp. in the case of clusterin) would add mucht to the paper.

# Minor

There seems to be some repetition between intro and results. Can perhaps reduce that to allow spaces for points made above.

 You wrote:

Note that this metric only considers edges of the tumor phylogeny on which mutations are gained and ignores all the edges on which mutations are lost.

Would be good to say why this is a good measure.

Fig 1, caption: please clarify that in (b), variants are both SNPs and SNVs. Also in (d), it would be good to clarify how this is different from the mutation matrix in (b). Perhaps call one observed and the other "inferred" or something like that?

You may want to explain in a sentence or two why SNPs are labeled 1 at the root and SNVs are labeled 0 at the root. It's clear once one thinks about it a bit but can be confusing to

some readers.

# Typos:

"a combinatorial characterization constrained k-Dollo phylogenies" "These cells were were not used"

"the 0 entries resulting losses "

Sentences like this read odd: "[27] provided an ..". Perhas use \citet?

"We generate a copy-number state for each node in the tree, as described in the “Methods” section."

"if picked uniformly at random between"

**Reviewer 2**

Review of "ConDoR: Tumor phylogeny inference with a copy-number constrained mutation loss model" submitted to Genome Biology.


SUMMARY:
In this work, Sashittal et al. introduce ConDoR, a method for reconstruction of trees of tumor evolution from single-cell sequencing data allowing mutational losses constrained with copy-number aberrations. In addition to copy number aberrations, ConDoR also leverages the information from germline variants (single-nucleotide polymporphism).

Tree reconstruction from single-cell sequencing data is an important problem that gained a lot of attention in the past decade. The first methods for solving this problem (e.g., SCITE and OncoNEM) operate under the infinite sites assumption, that is, each mutation can be gained at most once and once it is gained in some cell it is never lost in any of its descendants. Later methods (e.g., SPhyR or gpps) extended SCITE and OncoNEM by using k-Dollo parsimony model where each mutation can be lost up to k times. Finally, some methods (e.g., PhISCS) focus on identifying mutations for which infinite sites assumption is violated either due to losses or multiple gains of the same variant.

There are several mechanisms by which infinite sites assumption can be violated, but the vast majority of violations are expected to occur due do deletion events where large chromosomal regions are lost together with all SNVs that they harbor. Therefore, when for some mutation a loss is called by a method, a support in the matching copy number aberration data for such call is expected. Nevertheless, none of the existing methods takes advantage of this and they typically tend to overcall the number of variants for which infinite sites assumption is violated. ConDoR is, to the best of my knowledge, the first method which attempts to fill this gap. While, due to the way the clustering of cells is implemented and used during tree inference, in its implementation the method does not strictly enforce that SNV/SNP losses must be due to copy number aberrations, which I find as one of the main weaknesses, the Authors demonstrate that ConDoR can still have potential applications and yield novel insights in analyzing real clinical data. The Authors provide convincing evidence for advantage of ConDoR over the existing methods both on real and simulated data. On real data, they present evolutionary histories of Pancreatic ductal adenocarcinomas, which are well supported by data and more plausible than those obtined by existing alternatives, whereas on previously studied colorectal cancer dataset they report more parsimonious metastatic migration histories, which aligns with some previous analyses of this dataset (e.g., those presented in papers introducing PhISCS and SCARLET). On simulated datasets, it is shown that ConDoR outperforms the existing methods and consistently achieves higher accuracy in terms of two important and relevant accuracy measures.


RECOMMENDATION:
I find ConDoR a solid methodological contribution that could help researchers in studying the history of tumor evolution from single-cell sequencing data and I recommend its publication in Genome Biology, provided that my comments listed below are satisfactorily addressed.


MAJOR COMMENTS:

1.
In Definition 4, due to possible imperfect clustering of cells based on their copy number profiles, the second assumption might be violated (e.g., in cases where a cell harboring mutation is clustered within a cluster dominated by cells lacking the mutation due to a copy number loss). This can especially be the case when targeted sequencing data is used as the input since it might be challenging to distinguish homologous copies of chromosomal regions from such data. Robustness of ConDoR, at least on synthetic data, against the presence of this type of noise introduced during the cells clustering step needs to be demonstrated.

2.
When preparing the inputs to SCITE, some potentially stringent cutoffs (e.g. VAF of 0.10) were used to call mutations. Could the Authors please do the following:
(i) report the exact false positive and false negative error rates present in the inputs provided to SCITE on these simulated data.
(ii) Were the rates from (i) provided as priors when running SCITE?
(iii) Would SCITE benefit if the Authors put some additional efforts into improving/lowering these noise rates? For example, one possible potential reduction of false mutation calls might be achieved by creating pseudo-bulk samples by merging all reads from all simulated cells and leveraging information from those by going back and adjusting some mutation calls in individual cells.

3.
I would like to see how this method compares to SCITE when it comes to accuracy in cell-linege tree inference (i.e., when focus is placed on relative placement of cells instead of mutations). One possible measure of accuracy in this case is Robinson-Foulds, although it has known limitations (e.g., non-robustness to outliers in some cases) so the Authors might find some other measure more appropriate. Here, it is important to emphasize that I do not necessarily expect ConDoR to outperform SCITE in terms of this accuracy measure, but it is important to show that it is not significantly outperformed by SCITE. I do not expect this to happen, but I would prefer to see some empirical evidence for this.

4.
Please make sure that SCITE achieves full convergence. One option is to increase the number of repeats to 3 or 5. Alternatively, if you have sufficient time and computational resources, ILP-based methods that come with guarantees of optimality (e.g., PhISCS in ISA-mode, that is running PhISCS with -kmax 0) might be used either in addition to SCITE or to show that SCITE indeed finds optimal solution, provided appropriate and equivalent setting of noise parameters for both tools.

5. Was the ground truth clustering provided to ConDoR? If yes, please make sure that this is clearly indicated in the related section in Supplementary Materials.

Note that, although the above comments 2,3 and 4 in principle apply to all methods, I would be satisfied with comparisons against SCITE (which already performs on par with SCARLET in Figure 2b, and better than SPhyR).


MINOR COMMENTS:

In the definition provided on Page 3, second column, in lines 52-58 it should be precisely specified that there is a sort of bijection between the leaves of T and rows of A. The way it is currently written (especially in the last two of these lines), does not require/guarantee this.

In order to decompose the probability in Equation (1), it is necessary to assume that cells and mutations are sequenced independently, but also that they evolve independently. While the former assumption is stated in the paragraph preceding this equation, the latter is not. Please either add it or, if you disagree, explain in the Response to Reviewers why it is not necessary.

I find the first sentence in the paragraph starting on line 60, first column, Page 3 misleading. Namely, in the preceding paragraph, while defining violations of the infinite sites assumption, the Authors say that any loss of a mutation (regardless of whether it is due to reverse mutation or due to copy number aberrations) is not allowed under this assumption. However, in the sentence starting on line 60 it is first stated that violations of the infinite sites assumption are rare in cancer, but then the second part of the same sentence contradicts this by mentioning that losses are frequent. I assume that the Authors want to state that parallel and reverse mutations are rare. In addition to clarifying this, I also recommend providing a supporting reference for the claim (e.g., https://doi.org/10.1038/s41588-021-01005-8, which is already added as reference 23, or some other).

Due to the introduction of germline variants (SNPs), Definition 2 is not strictly identical to the definition from El-Kebir 2018 (I assume it is supposed to match Definition 6 from that paper, with some swaps in matrix names). This should be indicated before Definition 2 (e.g., by writing " ... the basis of the following (extended) definition of k-completion of a mutation matrix A."

Why are the variables g_{i,j}^{(0)} and g_{i,j}^{(1)}, defined on Page 10, continuous? There is not necessarily anything wrong with defining them as continuous, but aren't these variables binary since they can take only values at the boundary/endpoints of the segment [0,1]? The same comment applies to variables g_{i,j} defined on the same page.

Why is it used "We trim", "We assign" etc. when describing the pipeline in the first paragraph, left column, Page 13? Isn't this Mission Bio's commercial pipeline so the use of "we" might be misleading when used within this manuscript?

When checking PDAC_panel.xlsx file, I noticed that amplicon_number columns of some amplicons repeat. For example, there are the following two rows in that file:
chr17 7579314 7579536 AMPL9336 7579309 7579339 HV941444
chr17 7579314 7579536 AMPL9336 7579309 7579592 TP53
Also, the exonic segment [7579309,7579339] from the first of the above two rows the subset of the segment given by the second row. There is also the following amplicon with negative coordinates.
chr17 7576479 7576679 AMPL115428 -1 -1.
Can the Authors please comment on these? Is there some reference that can help with interpreting this file? If not, then adding one separate sheet (to the existing excel file) containing explanation of what each column means could perhaps clarify and answer all of these.

SiFit should be removed from the legend in Figure 2b since it had to be excluded from pairwise ancestral relation accuracy comparisons.

I could not understand the sentence "We assign reads to individual barcodes, filters for high-quality barcodes." on Page 13.

Please be more precise in the last sentence in subsection "Pairwise ancestral relationship accuracy" on Page 12.

Regarding the Reference 41: https://www.biorxiv.org/content/10.1101/2022.03.06.483206v1
- it seems that this paper has now been published so the reference should be updated.

In addition to the above, references 15 and 41 point to the same paper.


TYPOS AND SOME OTHER MINOR COMMENTS:
- On Page 3, first column, line 30 a binary matrix A is introduced. It is not clear why a_i,j is used to refer to the entries of this matrix, instead of A_i,j. This should be clarified.
- In the formula for computing normalized mutation matrix error (Page 4, second column), a'_i,j is used, which does not match the notation of A^=[a^_i,j}] introduced to denote a matrix inferred by a tool.
- Page 3, first column, line 55: multiple time -> multiple times
- Page 3, first column, lines 61 and 62: to due -> due to
- Page 3, second column, line 10: revisit "... but be lost ..."
- Page 3, second column, line 57: "... each leaf is labeling ..."
- (optional) Page 4, second column, lines 38-39 consider rewriting "which is used by all methods except SiFit in this study" into something like "which is employed (or used) by all methods that we compare against, except SiFit".
- Page 4, second column, line 46: "... because it a finite-site model ..."
- Page 4, second column, line 48: there should not be "," after "which".
- Page 7: "These cells were were not used ..."
- Page 9, first column, line 9: by -> but
- Page 9, first column, lines 11 and 12: " 0 entries resulting losses" should be slightly updated. Maybe by saying " 0 entries resulting from losses" or something like that.
- Page 9, first column, line 16: "." missing at the end of the sentence.
- Page 10, "." before the constraint on the sum of g_{l,j} is redundant.
- Page 13, first column, line 20: is there a typo in "gVCF's" ?
- Reference 57 contains "???".

**Authors’ response to reviewers**

# REVIEWER 1

Sashittal et al. propose a new method to infer tumor phylogeny while taking into account SNPs, SNVs, and CNAs. The approach is based on a new problem that defines an ILP-based solution.

The paper reads very well, is mostly very clear, and addresses an impactful problem. Most assumptions are very clear and the model appears sensible. The performance of the method on the real data seems very promising. In general, I have high enthusiasm for this paper. Below, I present several questions and suggestions. Some of these are best addressed using new experiments but I don't believe authors can be expected to perform those experiments (which are nontrivial). However, I do strongly suggest to the authors that they consider performing these additional experiments.

**Conceptual Questions**

I had several conceptual questions.

**Comment 1**

In the likelihood function (1) I see no term that penalizes a mutation. That is, we never go over branches and assess the likelihood of a mutation. I understand the problem is constrained by other things (e.g., the Dolo constraints). But again, there is no explicit term that penalizes more/less mutations. Two scenarios, with different numbers of mutations, but exactly the same read count likelihood would be in a tie. Don't we need to choose the one with fewer mutations? If I am misunderstanding, please clarify. If this is by choice, please elaborate on why. If this is a limitation, please mention it (perhaps as future work).

Typically, phylogeny inference is done only using mutations that are detected in the cells using some existing variant calling method. As such, each mutation is assumed to have occurred in at least one cell. This is a standard assumption in almost all tumor phylogeny inference methods, including SCITE (Jahn et al. 2016, *Genome Biology*), SiFit (Zafar et al. 2017, *Genome Biology*), SPhyR (El-Kebir 2018 *Bioinformatics*) and SCARLET (Satas *et al.* 2020 *Cell Systems*). Further, under the constrained k-Dollo model, a mutation occurs at most once in the phylogeny. As such, we do not need to penalize the number of mutations in the solution.

There are other methods that simultaneously perform variant calling and phylogeny inference, such as SciPhi (Singer et al. Nature Communications 2018; <https://www.nature.com/articles/s41467-018-07627-7>). ConDoR can be extended in the future to similarly perform variant calling and phylogeny inference simultaneously. We include this direction of research as future work in the Conclusions as follows.

*“First, ConDoR currently takes the read count matrices with the mutations and the copy-number clustering as input to build a constrained k-Dollo phylogeny. A future direction is to extend*

*ConDoR to simultaneously perform variant calling, and inference of the copy-number clustering and the phylogeny, potentially improving the accuracy of the results.”*

**Comment 2**

(small point) I assumed that copy-number clustering is considering all CNAs across the genomes and summarizing them into a single clustering. Would be good to say this clearly upfront.

We changed the text to make this clear in the first paragraph of the results section (in subsection ‘Constrained k-Dollo model’). Specifically, we add the following text.

*“Our model incorporates CNAs via a clustering of cells, where all cells in the same cluster have the same copy-number profile (the copy number of all loci across the genome). In other words, each cluster corresponds to a* copy-number clone*. We will refer to this clustering as the* copy-number clustering*.”*

**Comment 3**

A shortcoming of the paper is that the method relies on two assumptions (which is fine) but there is no in-depth discussion of those assumptions

- Assumption 1) In particular, you need to explain why the CNV losses are allowed only among different copy-number clusters. We need a bit more biological mechanistic justification here. On the other hand, if there is no biological reason and the choice is mostly to ease computations, that's fine too, but it needs to be stated. This assumption seems fundamental and deserves more room.

There are two ways in which a mutation can be *lost* in cancer. First, a *back mutation* can occur, i.e. the variant allele reverts back to the wildtype allele. Second, the mutation can be lost due to an overlapping copy number deletion. It has been shown in Kuiper et al. 2017 (<https://genome.cshlp.org/content/27/11/1885>) that since copy number aberrations (CNAs) are common in cancer, copy number deletions are a more likely explanation for loss of mutations compared to back mutations. This observation was also supported by simulations performed in a recent study by Demeulemeester et al. (<https://www.nature.com/articles/s41588-021-01005-8>) that investigated violations of the infinite sites assumption in cancer.

We cite the above references to make this reasoning more clear in the main text, as follows.

*“First, since reversal of mutations (back mutations) in cancer are rare (Kuiper et al.,* Demeulemeester et al.*), we assume that SNVs and SNPs are lost only via CNAs. As such, we only allow such losses between cells that belong to distinct copy-number clusters.”*

- Assumption 2) I would like to hear a bit more about why clusters of copy-numbers should arise only once. For one region, I don't see how this is reasonable. But perhaps clustering across the genome makes this reasonable. I am pretty sure there is a reason but more discussion is needed.

We agree that for an individual genomic locus it is unreasonable to assume that a copy number state is attained at most once in the phylogeny. Instead we assume that the copy number *profile* describing the copy number states over the entire genome arises at most once in the phylogeny.

We changed the main text to explain this more clearly as follows.

*“Second, we assume that each copy-number profile describing the copy number states over the entire genome arises only once in the phylogeny. As such, cells belonging to the same cluster form a connected subtree in the phylogeny.”*

**Experimental Results Comment 1**

I am only mildly enthusiastic about the simulation results. The reason is that the authors have

simulated under the exact set of assumptions provided and then have shown that the method works. This tells me relatively little. It's valuable but it doesn't tell me if the method is robust to violations of the assumptions. In my mind, the ultimate test is whether the model is good, not whether the method is able to maximize the score defined under the model.

- In particular, it seems the method is given the correct copy number clusters. What if those are wrong?
- Would the most be robust if the counts are not drawn from the negative binomial distribution?

**Suggestion**: For (1), my suggestion is that the authors use a simulated dataset from a previous publication, probably not fully compatible with their model, to also test their data. To summarize, experiments that show robustness to model violations and incorrect input (esp. in the case of clustering) would add much to the paper.

We follow the reviewer’s suggestion and compare the performance of ConDoR with existing methods on simulated dataset from a previous publication, i.e. from Satas et al. 2020 Cell Systems (<https://www.sciencedirect.com/science/article/pii/S2405471220301150>). This simulation dataset consists of 50 instances, each with 100 cells and 20 mutations. Further, we evaluate the robustness of ConDoR to varying levels of noise in the copy number clusters of these simulation instances. We add a detailed explanation of the simulations and describe these results in Section D of the supplement. We briefly summarize the results here.

To address the reviewer’s question about the sensitivity of the simulation results with respect to the read count model, we use the mutation matrices and trees simulated by Satas et al. 2020 and then perform comparisons in two different settings:

1. When the read counts are simulated using the negative binomial model described in our manuscript.
2. When the read counts are simulated using the binomial model described in Satas et al. 2020.

The results on the first set of simulations is shown in Figure S2, where the read counts are simulated using the negative binomial model. Similar to our simulation results, ConDoR and SCARLET are the top performing methods. We see that ConDoR has very similar performance to SCARLET (median 0.007 and 0.0065, respectively) in terms of the normalized mutation matrix error while the pairwise ancestral relation accuracy of ConDoR is higher than SCARLET (0.87 vs 0.84). We emphasize that SCARLET requires more information in input compared to ConDoR; namely SCARLET requires a copy number *tree* while ConDoR requires only a clustering of cells.

**(a)	(b)**

Figure S2: (a) Normalized mutation matrix error and (b) pairwise ancestral relation accuracy for each method on simulations from Satas et al. 2020, where the read counts were simulated using the negative binomial model.

**(a)	(b)**

Figure S3: (a) Normalized mutation matrix error and (b) pairwise ancestral relation accuracy for each method on simulations from Satas et al. 2020, where the read counts were simulated using the binomial model.

Figure S3 shows the results when the read counts are simulated using the binomial model described in Satas et al. 2020. Under this setting, ConDor outperforms all the methods except SCARLET. While the pairwise ancestral relation accuracy of SCARLET and ConDoR is very similar (median 0.8578 and 0.8578, respectively), we find that SCARLET outperforms ConDoR (median 0.0145 vs 0.0185) in terms of the mutation matrix error. However, this is expected because SCARLET is given the copy number clustering and the ground-truth copy number tree as input while ConDoR is only given the copy number clustering as input. Moreover, we use the same read count model to generate these simulations as the one used by SCARLET during inference.

Next, we evaluate the robustness of ConDoR when cells are assigned to the wrong copy

number clusters. Let

ℎ ∈ {0. 05, 0. 1, 0. 15, 0. 2}

be the fraction of cells for which we provide

incorrect cluster information. We simulate 5 instances for each value of *h*. Figure 3 shows that ConDoR is robust to errors in the copy number clusters. In fact, even when 20% of the cells have incorrect copy number state, ConDoR has lower normalized mutation matrix error (median 0.044) and higher pairwise ancestral relation accuracy (median 0.774) compared to SPhyR (0.046 and 0.737), SiFit (0.051 and N/A) and SCITE (0.044 and 0.705).

**(a)	(b)**

Figure S4: (a) Normalized mutation matrix error and (b) pairwise ancestral relation accuracy of ConDoR for increasing fraction of cells with incorrect copy number clusters in simulated instances. Dashed lines show the median performance of the other methods.

**Minor Comments**

- There seems to be some repetition between intro and results. Can perhaps reduce that to allow spaces for points made above

Thank you for the suggestion. We shortened the last two paragraphs of the Introduction to remove redundancy with the Results.

- You wrote: ‘Note that this metric only considers edges of the tumor phylogeny on which mutations are gained and ignores all the edges on which mutations are lost.’

Would be good to say why this is a good measure.

We changed the text to specify that when pairwise ancestral relation accuracy E(T, T’) is 1, then the two tumor phylogenies T and T’ are *identical* when restricted to the edges on which mutations are gained.

*“When the pairwise ancestral relation accuracy E(T, \hat{T}) is 1, the inferred tumor phylogeny \hat{T} and the ground-truth tumor phylogeny T are identical when restricted to the edges on which the mutations are gained.”*

- Fig 1, caption: please clarify that in (b), variants are both SNPs and SNVs. Also in (d), it would be good to clarify how this is different from the mutation matrix in (b). Perhaps call one observed and the other "inferred" or something like that?

We changed the caption to specify that the matrices in (b) are observed and the mutation matrix in (d) is inferred.

- You may want to explain in a sentence or two why SNPs are labeled 1 at the root and SNVs are labeled 0 at the root. It's clear once one thinks about it a bit but can be confusing to some readers.

To make it clearer for the readers, we changed the text to indicate that SNPs are germline mutations and SNVs are somatic mutations.

*“The root r(T) of the tree T represents the normal cell. As such a_{r(T), j} = 0 if mutation j is an SNV, i.e. a somatic mutation, and a_{r(T), j} = 1 if mutation j is an SNP, i.e. a germline mutation.”*

**Typos**

- "a combinatorial characterization constrained k-Dollo phylogenies"
- "These cells were were not used"
- "the 0 entries resulting losses "
- Sentences like this read odd: "[27] provided an ..". Perhaps use \citet?
- "We generate a copy-number state for each node in the tree, as described in the “Methods” section."
- "if picked uniformly at random between"

We fixed these and other typos we found after proof-reading the manuscript.

# REVIEWER 2

In this work, Sashittal et al. introduce ConDoR, a method for reconstruction of trees of tumor evolution from single-cell sequencing data allowing mutational losses constrained with copy-number aberrations. In addition to copy number aberrations, ConDoR also leverages the information from germline variants (single-nucleotide polymorphism).

Tree reconstruction from single-cell sequencing data is an important problem that gained a lot of attention in the past decade. The ﬁrst methods for solving this problem (e.g., SCITE and OncoNEM) operate under the inﬁnite sites assumption, that is, each mutation can be gained at most once and once it is gained in some cell it is never lost in any of its descendants. Later methods (e.g., SPhyR or gpps) extended SCITE and OncoNEM by using k-Dollo parsimony model where each mutation can be lost up to k times. Finally, some methods (e.g., PhISCS) focus on identifying mutations for which the inﬁnite sites assumption is violated either due to losses or multiple gains of the same variant.

There are several mechanisms by which inﬁnite sites assumption can be violated, but the vast majority of violations are expected to occur due to deletion events where large chromosomal regions are lost together with all SNVs that they harbor. Therefore, when for some mutation a loss is called by a method, a support in the matching copy number aberration data for such a call is expected. Nevertheless, none of the existing methods takes advantage of this and they typically tend to overcall the number of variants for which inﬁnite sites assumption is violated. ConDoR is, to the best of my knowledge, the ﬁrst method which attempts to ﬁll this gap. While, due to the way the clustering of cells is implemented and used during tree inference, in its implementation the method does not strictly enforce that SNV/SNP losses must be due to copy number aberrations, which I ﬁnd as one of the main weaknesses, the Authors demonstrate that ConDoR can still have potential applications and

yield novel insights in analyzing real clinical data. The Authors provide convincing evidence for the advantage of ConDoR over the existing methods both on real and simulated data. On real data, they present evolutionary histories of Pancreatic ductal adenocarcinomas, which are well supported by data and more plausible than those obtained by existing alternatives, whereas on previously studied colorectal cancer dataset they report more parsimonious metastatic migration histories, which aligns with some previous analyses of this dataset (e.g., those presented in papers introducing PhISCS and SCARLET). On simulated datasets, it is shown that ConDoR outperforms the existing methods and consistently achieves higher accuracy in terms of two important and relevant accuracy measures.

**Recommendations**

I ﬁnd ConDoR a solid methodological contribution that could help researchers in studying the history of tumor evolution from single-cell sequencing data and I recommend its publication in Genome Biology, provided that my comments listed below are satisfactorily addressed.

## Major Comments Comment 1

In Deﬁnition 4, due to possible imperfect clustering of cells based on their copy number

proﬁles, the second assumption might be violated (e.g., in cases where a cell harboring mutation is clustered within a cluster dominated by cells lacking the mutation due to a copy number loss). This can especially be the case when targeted sequencing data is used as the input since it might be challenging to distinguish homologous copies of chromosomal regions from such data. Robustness of ConDoR, at least on synthetic data, against the presence of this type of noise introduced during the cells clustering step needs to be demonstrated.

We received a similar comment from Reviewer 1 about evaluating the robustness of ConDoR when cells are assigned to the wrong copy number clusters. Please refer to the results of this analysis in Figure S4. We add a detailed explanation of the simulations and describe the results in Section D of the supplement. In brief, ConDoR is robust to inaccuracies in the copy number clustering and outperforms SCITE, SiFit and SPhyR even when a significant number of cells are assigned the wrong copy number clusters. Specifically, even when 20% of the cells have incorrect copy number state, ConDoR has lower normalized mutation matrix error (median 0.044) and higher pairwise ancestral relation accuracy (median 0.774) compared to SPhyR (0.046 and 0.737), SiFit (0.051 and N/A) and SCITE (0.044 and 0.705).

## Comment 2

When preparing the inputs to SCITE, some potentially stringent cutoffs (e.g. VAF of 0.10) were used to call mutations. Could the Authors please do the following:

(i) report the exact false positive and false negative error rates present in the inputs provided to SCITE on these simulated data.
(ii) Were the rates from (i) provided as priors when running SCITE?
(iii) Would SCITE beneﬁt if the Authors put some additional efforts into improving/lowering these noise rates? For example, one possible potential reduction of false mutation calls might be achieved by creating pseudo-bulk samples by merging all reads from all simulated cells and leveraging information from those by going back and adjusting some mutation calls in individual cells.
(i) We report the false positive and false negative rates in the simulated data in Figure S1 of Section C of the supplement. The median values for the false positive and false negative rates are 0.0018 and 0.158, respectively.
(ii) The median of the false positive and false negative rates were provided as priors when running SCITE, SiFit and SPhyR. The parameters and precise commands used to run each method on the simulated instances are described in Section C of the supplement.
(iii) We agree with the reviewer that employing more elaborate techniques to prepare input for SCITE might lead to improvement in the results produced by SCITE. However, these techniques are not standard and also not part of the SCITE algorithm. The false positive and false negative rates based on our approach of obtaining the observed mutation matrix are similar to the values observed in real data. For instance, for the metastatic colorectal cancer dataset from Leung *et al.* 2017, the authors suggested a false positive rate of 0.0174 and false negative rate of 0.1256.

To make it clear that there is room for improvement in the way observed mutation matrices in the main text, we add the following sentence in the subsection *‘Obtaining the observed mutation matrix from the read counts*’

*“The median false positive and false negative rates of the observed mutation matrices of the simulated instances are 0.0018 and 0.158, respectively (Additional File: Figure S1). It is possible that using additional procedures to define a higher quality set of mutations could improve the performance of methods such as SPhyR, SCITE and SiFit.”*

## Comment 3

I would like to see how this method compares to SCITE when it comes to accuracy in cell-lineage tree inference (i.e., when focus is placed on relative placement of cells instead of mutations). One possible measure of accuracy in this case is Robinson-Foulds, although it has known limitations (e.g., non-robustness to outliers in some cases) so the Authors might ﬁnd some other measure more appropriate. Here, it is important to emphasize that I do not necessarily expect ConDoR to outperform SCITE in terms of this accuracy measure, but it is important to show that it is not signiﬁcantly outperformed by SCITE. I do not expect this to happen, but I would prefer to see some empirical evidence for this.

We add the Robinson-Foulds distances of the simulated phylogenies from the inferred phylogenies of each method in Figure 5a in the Supplement. We find that ConDoR has the lowest distance (median 0.31) from the ground-truth, indicating that it infers the most accurate cell lineage tree compared to the existing methods (SCARLET: 0.36, SPhyR: 0.86, SiFit: 0.76 and SCITE: 0.55).

Figure S5(a): Normalized Robinson-Foulds distance of the phylogenies inferred by all the methods from the ground-truth phylogeny.

## Comment 4

Please make sure that SCITE achieves full convergence. One option is to increase the number of repeats to 3 or 5. Alternatively, if you have suﬃcient time and computational resources, ILP-based methods that come with guarantees of optimality (e.g., PhISCS in ISA-mode, that is running PhISCS with -kmax 0) might be used either in addition to SCITE or to show that SCITE indeed ﬁnds optimal solution, provided appropriate and equivalent setting of noise parameters for both tools.

We repeated the simulations by running SCITE by setting the number of repeats parameters to

5. The results in the main text have been updated. We see that the performance of ConDoR is still better than SCITE on the simulated instances, both in terms of normalized mutation matrix error (median 0.0038 vs 0.0156) and pairwise ancestral relation accuracy (median 0.98 vs 0.967).

## Comment 5

Was the ground truth clustering provided to ConDoR? If yes, please make sure that this is clearly indicated in the related section in Supplementary Materials.

Yes, we provide the ground-truth copy number clustering to ConDoR as input. In the same vein, SCARLET is given the ground-truth copy number clustering and the copy number tree as input. We indicate this in the supplementary Section C as follows.

*Note that ConDoR is provided the accurate copy number clusters in the simulations as input, and SCARLET is provided the accurate copy number clusters and the ground-truth copy number tree in the simulations as input.*

Note that, although the above comments 2,3 and 4 in principle apply to all methods, I would be satisﬁed with comparisons against SCITE (which already performs on par with SCARLET in Figure 2b, and better than SPhyR).

## Minor Comments Comment 1

In the deﬁnition provided on Page 3, second column, in lines 52-58 it should be precisely

speciﬁed that there is a sort of bijection between the leaves of T and rows of A. The way it is currently written (especially in the last two of these lines), does not require/guarantee this.

We have now changed the phrasing to convey that there must be a bijection between the leaves of T and rows of A. Specifically, we now say that for each leaf there must be a *unique* index i such that the leaf is labeled by ith row of A and \sigma(i).

## Comment 2

In order to decompose the probability in Equation (1), it is necessary to assume that cells and mutations are sequenced independently, but also that they evolve independently. While the former assumption is stated in the paragraph preceding this equation, the latter is not. Please either add it or, if you disagree, explain in the Response to Reviewers why it is not necessary.

Yes, we agree with the reviewer. When the phylogeny is given, the cells and mutations are sequenced and *evolve* independently. We have changed the text to reflect this as follows.

*Assuming that the cells and mutations in each cell are sequenced independently, and that given the tree, the cells evolve independently, the likelihood of observing …*

**Comment 3**

I ﬁnd the ﬁrst sentence in the paragraph starting on line 60, ﬁrst column, Page 3 misleading. Namely, in the preceding paragraph, while deﬁning violations of the inﬁnite sites assumption, the Authors say that any loss of a mutation (regardless of whether it is due to reverse mutation or due to copy number aberrations) is not allowed under this assumption. However, in the sentence starting on line 60 it is ﬁrst stated that violations of the inﬁnite sites assumption are rare in cancer, but then the second part of the same sentence contradicts this by mentioning that losses are frequent. I assume that the Authors want to state that parallel and reverse mutations are rare. In addition to clarifying this, I also recommend	providing	a	supporting	reference	for	the	claim (e.g., [https://doi.org/10.1038/s41588-021-01005-8](https://nam12.safelinks.protection.outlook.com/?url=https%3A%2F%2Fdoi.org%2F10.1038%2Fs41588-021-01005-8&data=05%7C01%7Cps7731%40princeton.edu%7C155aee6a008f4b771adf08db3aca20fe%7C2ff601167431425db5af077d7791bda4%7C0%7C0%7C638168412040875473%7CUnknown%7CTWFpbGZsb3d8eyJWIjoiMC4wLjAwMDAiLCJQIjoiV2luMzIiLCJBTiI6Ik1haWwiLCJXVCI6Mn0%3D%7C3000%7C%7C%7C&sdata=Vc0bVuqu4ZsBpPW8OGOF4HlQNppAv8U9jiP0nnJ%2FNVk%3D&reserved=0), which is already added as reference 23, or some other).

We thank the reviewer for pointing this out and the helpful suggestion of supporting reference. We use the suggested wording of ‘parallel and reverse mutations’ and add the reference in the text as follows.

*While parallel mutations (i.e. the same mutation gained multiple times in the phylogeny) and back mutations (i.e. a mutation reverted back to reference state) are rare in cancer (Demeulemeester et al.), SNVs and SNPs are frequently lost due to copy-number aberrations.*

**Comment 4**

Due to the introduction of germline variants (SNPs), Deﬁnition 2 is not strictly identical to the deﬁnition from El-Kebir 2018 (I assume it is supposed to match Deﬁnition 6 from that paper, with some swaps in matrix names). This should be indicated before Deﬁnition 2 (e.g., by writing " ... the basis of the following (extended) deﬁnition of k-completion of a mutation matrix A."

We have replaced the text in the manuscript with the suggested text.

**Comment 5**

Why are the variables g_{i,j}^{(0)} and g_{i,j}^{(1)}, deﬁned on Page 10, continuous? There is not necessarily anything wrong with deﬁning them as continuous, but aren't these variables binary since they can take only values at the boundary/endpoints of the segment [0,1]? The same comment applies to variables g_{i,j} deﬁned on the same page.

These variables are defined as continuous because the enforced constraints in the ILP formulation will always make sure that these variables can only attain integer values, i.e. either 0 or 1. Defining the variables as continuous leads to improvement in the performance of the ILP solver.

**Comment 6**

Why is it used "We trim", "We assign" etc. when describing the pipeline in the ﬁrst paragraph, left column, Page 13? Isn't this Mission Bio's commercial pipeline so the use of "we" might be misleading when used within this manuscript?

Yes, we use Mission Bio’s commercial pipeline. We reworded these sentences to avoid confusion.

**Comment 7**

When checking PDAC_panel.xlsx ﬁle, I noticed that amplicon_number columns of some amplicons repeat. For example, there are the following two rows in that ﬁle:

chr17 7579314 7579536 AMPL9336 7579309 7579339 HV941444 chr17 7579314 7579536 AMPL9336 7579309 7579592 TP53

Also, the exonic segment [7579309,7579339] from the ﬁrst of the above two rows the subset of the segment given by the second row. There is also the following amplicon with negative coordinates.

chr17 7576479 7576679 AMPL115428 -1 -1.

Can the Authors please comment on these? Is there some reference that can help with interpreting this ﬁle? If not, then adding one separate sheet (to the existing excel ﬁle) containing an explanation of what each column means could perhaps clarify and answer all of these.

We thank the reviewer for the careful review of our supplementary panel design sheet.

In order to query how well our targeted panel covers the exonic regions of our genes of interest, we ran `bedtools intersect` on the panel insert file against the hg19 canonical exons downloaded from UCSC, which simply found the intersection of the two’s genomic regions. The panel insert file’s original columns are the first four columns of PDAC_panel.xlsx, while the UCSC hg19 canonical exons file’s original columns are the last three columns.

To answer the first question: for the hg19 canonical exons downloaded from UCSC, each unique genomic region (e.g. chr17:7579309-7579339) could map to different genes and exons (e.g. HV941444 and TP53).

To answer the second question: some amplicons, AMPL115428 being an example, do not intersect with any hg19 canonical exon. Hence bedtools will output “-1” and “.” to the last three columns. These amplicons, however, either map to non-canonical exons which would still transcribe as alternate mRNA transcripts, or map to non-exonic regions of genes of interest.

We recognize that the original PDAC_panel.xlsx file provided no explanation as above, so we added a separate sheet as description to the file. The updated excel sheet has been uploaded on github at the following link.

https://github.com/raphael-group/constrained-Dollo/blob/main/data/PDAC/PDAC_panel.xlsx

**Comment 8**

SiFit should be removed from the legend in Figure 2b since it had to be excluded from pairwise ancestral relation accuracy comparisons.

We remove SiFit from the legend of Figure 2b and all other figures in the manuscript where pairwise ancestral relation accuracy between different methods is compared.

## Comment 9

I could not understand the sentence "We assign reads to individual barcodes, ﬁlters for high-quality barcodes." on Page 13.

We fixed this typo.

**Comment 10**

Please be more precise in the last sentence in subsection "Pairwise ancestral relationship accuracy" on Page 12.

We made the heading of the subsection and the description more clear.

**Comment 11**

Regarding	the	Reference	41:

[https://www.biorxiv.org/content/10.1101/2022.03 06.483206v1](https://nam12.safelinks.protection.outlook.com/?url=https%3A%2F%2Fwww.biorxiv.org%2Fcontent%2F10.1101%2F2022.03.06.483206v1&data=05%7C01%7Cps7731%40princeton.edu%7C155aee6a008f4b771adf08db3aca20fe%7C2ff601167431425db5af077d7791bda4%7C0%7C0%7C638168412040875473%7CUnknown%7CTWFpbGZsb3d8eyJWIjoiMC4wLjAwMDAiLCJQIjoiV2luMzIiLCJBTiI6Ik1haWwiLCJXVCI6Mn0%3D%7C3000%7C%7C%7C&sdata=zvy9Qv%2FZgOUqal09slOlsDMpv%2BmDoGOOHAjEKav%2FHuM%3D&reserved=0)

- it seems that this paper has now been published so the reference should be updated. In addition to the above, references 15 and 41 point to the same paper.

We thank the reviewer for catching this. We have fixed the aforementioned references.

**Typos and some other minor comments**

- On Page 3, ﬁrst column, line 30 a binary matrix A is introduced. It is not clear why a_i,j is used to refer to the entries of this matrix, instead of A_i,j. This should be clariﬁed.

We have been consistent with this style of notation. We use capital letters for matrices and small letters for elements of the matrices.

- In the formula for computing normalized mutation matrix error (Page 4, second column), a'_i,j is used, which does not match the notation of A^=[a^_i,j}] introduced to denote a matrix inferred by a tool.
- Page 3, ﬁrst column, line 55: multiple time -> multiple times
- Page 3, ﬁrst column, lines 61 and 62: to due -> due to
- Page 3, second column, line 10: revisit "... but be lost	"
- Page 3, second column, line 57: "... each leaf is labeling	"
- (optional) Page 4, second column, lines 38-39 consider rewriting "which is used by all methods except SiFit in this study" into something like "which is employed (or used) by all methods that we compare against, except SiFit".
- Page 4, second column, line 46: "... because it a ﬁnite-site model "
- Page 4, second column, line 48: there should not be "," after "which".
- Page 7: "These cells were were not used	"
- Page 9, ﬁrst column, line 9: by -> but
- Page 9, ﬁrst column, lines 11 and 12: " 0 entries resulting losses" should be slightly updated. Maybe by saying " 0 entries resulting from losses" or something like that.
- Page 9, ﬁrst column, line 16: "." missing at the end of the sentence.
- Page 10, "." before the constraint on the sum of g_{l,j} is redundant.
- Page 13, ﬁrst column, line 20: is there a typo in "gVCF's" ?
- Reference 57 contains "???".

We fixed these and other typos we found after proof-reading the manuscript.

**Second round of review**

**Reviewer 1**

Authors have addressed all my comments in the revision. In particular, I find the addition of Figure S4 quite useful. In fact, I encourage the authors to *consider* moving that figure (or some parts of it) to the main paper as I imagine users of the methods will wonder about robustness (both reviewers did) and having the figure in the main paper would make it easier to note the trends.

**Reviewer 2**

Second Review of "ConDoR: Tumor phylogeny inference with a copy-number constrained mutation loss model" submitted to Genome Biology.


Recommendation:
Minor Revision


I thank the Authors for revising the manuscript and addressing my previous comments. I recommend minor revision of this work where the comments listed below should be addressed.

Major:

1. In order to facilitate the usage of ConDoR software, please improve description of its input and output by providing examples of the input files accompanied with more detailed description of what their rows and columns represent. Analogous applies to the output files.

2. Related to the previous point, the software would also greatly benefit if users are given some guidance on how to obtain input copy number clustering of cells.

3. When SNV/SNP losses occur, they are expected to be supported by copy number losses. There should not be losses between cells in clusters C1 and C2, where C1 is ancestral to C2 and C1 and C2 only differ by gains in C2. To be more precise, consider SNV/SNP M in gene g and assume that copy number cluster C1 has 1 maternal and 1 paternal copy of chromosomal segment containing g. Assume that M occurs for the first time in some of the cells of C1. Next, assume that C2 is another cluster such that cells in C2 have 2 maternal and 1 paternal copy of the segment containing g. There should be no losses of M permitted between C1 and C2. Are there constraints in the current formulation that enforce this?


Minor:

- In the tree shown in Figure 3b, mutation in gene BRCA2 is placed as a clonal and is present in two out of three orange nodes. But, according to the plot in panel d of the same figure, this mutation is mostly not observed in orange (C1) cluster. One potential explanation of this is that most of the cells in C1 cluster are healthy (normal) cells. Could you please comment on this in the main text? Showing the exact numbers of cells from each sample next to the tree nodes would be more informative than showing fractions (in that case such numbers should be added to the root node as well). Note that from such numbers, not just that the fractions currently shown could be trivially computed, but it would also enable readers to easily find several other statistics of interest (e.g., what fraction of all cells from sample 1 are clustered in green cluster etc.).

- In Results section, sigma is defined as mapping from [n] to [p], and later used in Definition 1 as if it was mapping from V(T) to [p]. Please explicitly indicate that this is done for the sake of notation convenience.

- Labels of x-axis in Figure 2a seem to be missing one dimension of the simulated matrices.

- The median length of 209 bps is provided for the panel used for targeted sequencing of PDAC samples. Please also provide average panel length or, alternatively, the total number of bps covered by the whole set of amplicons.

- In "Simulation of the phylogeny" section in Methods section, the last sentence is ambiguous when stating "as described in "Methods" section".

- In section "Simulation of copy-number states" the first sentence is not precise and needs to be updated.

- Check if there is a typo in "... as long as these losses to conform to a given copy-number clustering ...".

- Please revisit the sentence "The published study build a phylogeny of the 186 cells using SCITE ...". It contains a typo in "build" and, other than that, I believe that its readability can be slightly improved (e.g., it might more appropriate to use "original study" instead of "published study").

- "... in agreement with the PhISCS and SCARLET analysis": analysis -> analyses ?

- Figure 4, caption: "((bold text in (a))", redundant "(" in "((".

- Is there a typo in "copy-numbers profiles"? (Conclusions section)

**Authors’ response to reviewers**

We have revised the manuscript in response to the reviewer comments and we have also

addressed all editorial and formatting requests. In particular, we have made the following substantial

changes.

● Improved the description of the ConDoR software on the github repository.

● Added a data and code availability statement in accordance with the guidelines.
